# Increased expression of *SYCP2* predicts poor prognosis in patients suffering from breast carcinoma

**DOI:** 10.3389/fgene.2022.922401

**Published:** 2022-09-07

**Authors:** Hongyan Zheng, Xiaorong Guo, Nan Li, Luyao Qin, Xiaoqing Li, Ge Lou

**Affiliations:** ^1^ Department of Pathology, The Second Affiliated Hospital of Harbin Medical University, Harbin, China; ^2^ Department of Pathology, The Fourth Affiliated Hospital of Harbin Medical University, Harbin, China

**Keywords:** SYCP2, breast carcinoma, bioinformatics analysis, Gene Expression Omnibus database, survival analysis

## Abstract

Overexpression of synaptonemal complex protein-2 (*SYCP2*) has been identified in various human papillomavirus (HPV)–related carcinomas, whereas its significant role in breast carcinoma remains unclear. The aim of this study was to elucidate the prognostic value and potential function of *SYCP2* in breast carcinoma. Herein, data for breast carcinoma patients from the Gene Expression Omnibus (GEO) and The Cancer Genome Atlas database (TCGA) were analyzed. The enrichment analysis of *SYCP2* including Gene Ontology (GO), Kyoto Encyclopedia of Genes and Genomes (KEGG), Friends, and GSEA was performed. Kaplan–Meier analysis, Cox regression, and receiver operating characteristic (ROC) curves were employed for determining the predictive value of *SYCP2* on clinical outcomes in patients suffering from breast carcinoma. A nomogram was generated to predict the effect arising from *SYCP2* on prognosis. The association analysis of *SYCP2* gene expression and diverse immune infiltration levels was conducted through ssGSEA and ESTIMATE analysis, which consisted of dendritic cell (DC), neutrophil, eosinophil, macrophage, mast cell, NK cell, and other 18 cell subtypes. The results showed that *SYCP2* expression was significantly elevated in breast carcinoma tissues as compared with that of normal tissues (*p* < 0.001). *SYCP2* plays a certain role in pathways related to DNA methylation, keratinocyte differentiation, steroid hormone biosynthesis, and immune infiltration. The high expression of *SYCP2* had a significant relationship to age, pathological type, ER expression, and PR expression (*p* < 0.001). Kaplan–Meier survival analysis showed that patients suffering from breast carcinoma characterized by high-*SYCP2* expression had a poorer prognosis than patients with low-*SYCP2* expression (*p* = 0.005). Univariate and multivariate Cox regression analyses revealed that *SYCP2* had an independent relationship to overall survival (*p* = 0.049). Moreover, ROC curves suggested the significant diagnostic ability of *SYCP2* for breast carcinoma, and as time went on, *SYCP2* had more accurate prognostic efficacy. Furthermore, a high level of *SYCP2* expression was found to have a relationship to poor prognosis of breast carcinoma in the subgroups of T3, N0, and M0, and infiltrating ductal carcinoma (HR > 1, *p* < 0.05). The calibration plot of the nomogram indicated that the *SYCP2* model has an effective predictive performance for breast carcinoma patients. Conclusively, *SYCP2* plays a vital role in the pathogenesis and progression of human breast carcinoma, so it may serve as a promising prognostic molecular marker of poor survival.

## Introduction

The incidence rate of female breast carcinoma is 46.3% and the death rate is 13.0%, ranking first in female carcinoma, as reported by the international carcinoma research center and the American Carcinoma Society’s global carcinoma statistics report 2018 (Bray et al., 2018). It is a highly heterogeneous tumor with remarkable genetic and phenotypic diversity, as revealed in the proliferation rate, invasion ability, metastasis potential, therapeutic effect, and pathogenic mutation of tumor cells. At present, breast carcinoma has been largely treated by surgery, supplemented by radiotherapy, chemotherapy, and endocrine therapy. Treatments are capable of increasing the long-term cure rate of patients, whereas some of the treatment failures of breast carcinoma primarily arise from the high aggressiveness of the tumor and distant metastasis. Although tumor stage, histological grade, pathological classification, and immunophenotyping are generally applied in the prognosis clinically, the abovementioned features cannot accurately make the prognosis of patients due to tumor heterogeneity and the underlying pathogenesis of breast cancer aggressiveness which remains poorly understood. Accordingly, effective biomarkers for prognostic risk assessment and molecular targets should be identified for breast cancer treatment.

Synaptonemal complex protein-2 (SYCP2) is the largest synaptonemal complex (SC) protein yet described which consists of 1,530 amino acids in humans (Kouznetsova et al., 2005) and is the major component of the axial/lateral elements of SCs during meiotic prophase (Winkel et al., 2009; Fraune et al., 2014). Three major isoforms of SC proteins, including SC protein-1 (SYCP1), SC protein-2 (SYCP2), and SC protein-3 (SYCP3), were found to be the structural proteins of mammalian SCs. Thus, *SYCP2* plays a key role in the assembly of synaptonemal complexes and is required for normal meiotic chromosome synapsis during oocyte (Feng et al., 2017) and spermatocyte development (Takemoto et al., 2020).

In existing studies, *SYCP2* was reported as a robust candidate gene for male infertility since its encoding protein can interact directly with protein products of the male infertility genes *TEX11* and *SYCP3* in mice (Offenberg et al., 1998; Yang et al., 2008). Nevertheless, recent research provided further evidence of *SYCP2*-mediated male infertility, and reported that *SYCP2* translocation-mediated dysregulation and frameshift variants can result in human male infertility (Schilit et al., 2020). In addition, aberrant expression of *SYCP2* which has been considered as a testis-specific human gene was identified in human papillomavirus (HPV)–related tumors, including HPV-positive head and neck squamous cell carcinoma (HNSCC) and cervical squamous cell carcinoma. Concretely, an existing study observed that *SYCP2* was upregulated in HPV-positive HNSCC as compared with HPV-negative HNSCC (Martinez et al., 2007). Also, upregulated expression of *SYCP2* (Masterson et al., 2015) was revealed in premalignant tissue (e.g., oropharyngeal squamous cell carcinoma *in situ*). During the progression of cervical cancer, *SYCP2* was confirmed to be upregulated from normal cervical tissues, cervical intraepithelial neoplasia, to squamous cell carcinoma (Li et al., 2021). A recent study suggested that *SYCP2* was significantly upregulated in luminal B tumors compared with the adjacent normal tissues, and the upregulated *SYCP2* expression might serve as an independent indicator of shorter overall survival in luminal A/B breast carcinoma (Wu and Tuo, 2019). However, the potential role and relationship of *SYCP2* suffering from breast carcinoma have been rarely characterized.

**FIGURE 1:**
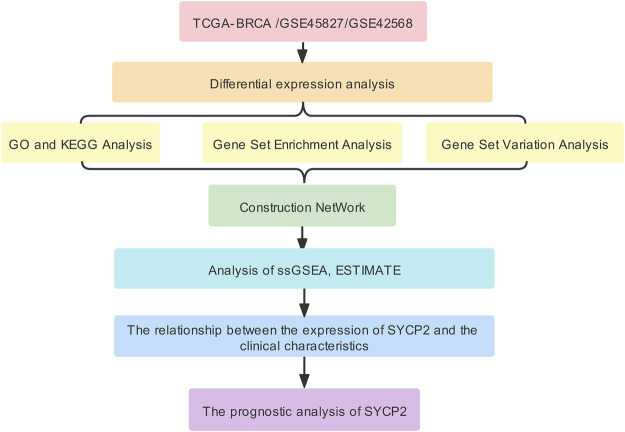
Workflow.

The aim of this study was to investigate differential mRNA expression of *SYCP2* and associated pathways. By performing functional and interaction network analysis, immune cell infiltration, clinicopathological correlation, and prognostic significance in patients suffering from breast carcinoma were determined using vastly increasing bioinformatics methods, applications, and databases to provide unique insights into the prognosis monitoring and treatment of breast carcinoma. The workflow of this study was shown in Figure 1.

## Materials and methods

### Data source

1,109 RNA-seq data (HTSeq-FPKM and HTSeq-counts) and corresponding clinical (Supplementary Data Sheets S1–S7) information of patients suffering from breast carcinoma originated from The Cancer Genome Atlas Breast Invasive Carcinoma (TCGA-BRCA) of the Genomic Data Commons (GDC) data portal (https://portal.gdc.cancer.gov/), which consisted of 112 breast carcinoma samples with matched adjacent tissues. The Gene Expression Omnibus (GEO, https://www.ncbi.nlm.nih.gov/geo/) refers to an open high-throughput sequencing gene expression database. The GSE45827 (Gruosso et al., 2016) (Supplementary Data Sheet S8) and GSE42568 (Clarke et al., 2013) (Supplementary Data Sheet S9) datasets originated from the GEO database, both of which were generated using the GPL570 (HG-U133_Plus_2) (Supplementary Data Sheet S10) Affymetrix Human Genome U133 Plus 2.0 Array. According to the GSE45827 dataset, 130 breast carcinoma tissues and 11 normal breast tissues were involved. In the GSE42568 dataset, 104 breast carcinoma tissues and 17 normal breast tissues were covered. The Human Protein Atlas (HPA) database (https://www.proteinatlas.org/) primarily involves proteomics, transcriptome, and systems biology data, providing tissue and cell distribution information of all 24,000 human proteins (Uhlen et al., 2015). In this study, protein expression data of SYCP2 in whole body tissues originated from the HPA database.

### Synaptonemal complex protein-2 differential expression in breast carcinoma tissues

Patients suffering from breast carcinoma were assigned to the high-*SYCP2* expression group and the low-*SYCP2* expression group in accordance with the *SYCP2* median value in the TCGA-BRCA database. Differentially expressed genes (DEGs) between the two groups were identified using the R package “DESeq2” (Love et al., 2014), in which the |logFC| >1 and Adjust *p*-Value< 0.05 were set as thresholds. The R packages “ggplot2” and “pheatmap” were adopted to illustrate results as volcano plots and heatmaps.

### Functional and pathway enrichment analysis

In accordance with the R package “clusterProfiler” (Yu et al., 2012), we performed functional enrichment analysis which included Gene Ontology (GO) terms for biological process (BP), cellular component (CC), and molecular function (MF) categories and Kyoto Encyclopedia of Genes and Genomes (KEGG) pathways. In order to identify the hub gene that interacts with other genes in the pathway, we used the R package “GOSemSim” (Yu et al., 2010) to conduct Friends analysis based on the GO analysis results. Only terms with a *p*-value < 0.05 were considered significant. Furthermore, gene set enrichment analysis (GSEA) was performed to elucidate the significant function and pathway differences between the high-*SYCP2* expression group and the low-*SYCP2* expression group. GSEA (Subramanian et al., 2005) is a computational method to analyze whether a particular gene set is statistically different between two biological states and is commonly used to estimate changes in pathway and biological process activity in expression dataset samples. The “C2. cp.v7.2.symbols. GMT (Curated)” gene set was downloaded from the Molecular Signatures Database (MSigDB) for GSEA analysis, and *p*-value< 0.05 was considered to be statistically significant enrichment. In addition, the R package “GSVA” (Hanzelmann et al., 2013) was used to calculate the score of the Hallmark pathway according to the gene expression matrix of the respective sample by the single-sample GSEA (ssGSEA) method (Barbie et al., 2009), and differential screening of enrichment function was performed by limma (Ritchie et al., 2015) package in R software. *p*-value < 0.05 was considered to be statistically significant.

### Construction of protein–protein interaction network

STRING (https://string-db.org/) refers to a user-friendly online system aiming at collecting, scoring, and integrating all publicly available sources of PPI data, as well as at complementing the aforementioned functions with computational predictions of potential functions (Szklarczyk et al., 2021). The PPI analysis of DEGs screened from the high-*SYCP2* expression group and the low-*SYCP2* expression group was performed through the STRING database, and the obtained results were visualized with the network analyzer tool of the Cytoscape software. The starBase database (Li et al., 2014) was used to search for microRNA targets on the basis of high-throughput CLIP-seq experimental data and degradation group experimental data, thus providing a wide variety of visualized interfaces to predict microRNA targets. This database covers considerable miRNA-ncRNA, miRNA-mRNA, RBP-RNA, and RNA-RNA data, and it can be predicted by multiple tools (PITA, RNA22, miRmap, DIANA-microT, miRanda, PicTar, and TargetScan) simultaneously for the search of miRNA–mRNA interactions. This study used the starBase database to predict miRNA and RNA-binding proteins (RBP) binding to *SYCP2*. Several parameters were set, including clade (mammal), genome (human), assembly (hg19), CLIP data (≥ 1), degradome data (≥ 0), pan-cancer (≥ 0), program num (≥ 1), program (none), and clade (mammal), genome (human), assembly (hg19), CLIP data (≥ 1), pan-cancer (≥ 0). In addition to humans, mice were included, and humans were selected for our current study. CLIP data indicates the number of sequencing data of crosslinking-immunoprecipitation. The higher the level, the higher the feasibility of the result, including low stringency (≥ 1), medium stringency (≥ 2), high stringency (≥ 3), and strict stringency (≥ 5). As there is no unified standard for these parameters, the parameters in the current study were selected according to the prediction results, as shown earlier. The PROMO platform is a virtual laboratory for studying the binding sites of transcription factors in DNA sequences (Messeguer et al., 2002; Farre et al., 2003). Through this platform, we predicted the transcription factors bound to *SYCP2*, where the maximum matrix dissimilarity rate was set to 5%. The Comparative Toxicogenomic Database (CTD) is a public database that links toxicological information on chemicals, genes, phenotypes, diseases, and exposures (Davis et al., 2021). For a specific gene, the CTD may predict the corresponding target compounds in a descending order of their interactions. In this study, CTDs with default parameters were used to provide the candidate chemicals associated with *SYCP2* genes. Lastly, the R package “igraph” was used to draw the interactive network graph.

### Immune infiltration analysis

Immune infiltration analysis of breast carcinoma samples was performed by the ssGSEA method using the R package “GSVA” (Hanzelmann et al., 2013) to assess the abundance of immune cells, which consisted of regulatory T cell (Treg), Th17 cell, Th2 cell, Th1 cell, neutrophil, eosinophil, macrophage, CD8 T cell, T-helper cell, T cell, NK CD56 bright cell, NK CD56 dim cell, B cell, NK cell, cytotoxic cell, mast cell, T-central memory (Tcm), T-effector memory (Tem), T-follicular helper (Tfh), T-gamma delta (Tgd), plasmacytoid DC (pDC), immature DC (iDC), activated DC (aDC), and dendritic cell (DC) in breast carcinoma samples. RNA-seq data (level-3 HTSeq-FPKM) were extracted from TCGA-BRCA. The relative enrichment score of each was quantified from the gene expression profile for each tumor sample. Default parameters in the package were used. ESTIMATE analysis refers to an algorithm that quantifies immune activity (the level of immune invasion) in this tumor sample in accordance with gene expression profiles. In this study, the immune activity and matrix score for the respective sample of breast carcinoma (level-3 HTSeq-FPKM) were assessed using the ESTIMATE package (Yoshihara et al., 2013) in R, and default parameters in the package were used.

### Correlation and prognosis analysis between synaptonemal complex protein-2 expression and clinicopathological characteristics

The time-dependent receiver operating characteristic (ROC) curve was performed according to the high- and low-*SYCP2* expression to evaluate the diagnostic efficacy of *SYCP2* in breast carcinoma. The prognostic value of *SYCP2* in breast carcinoma was assessed with the use of the Kaplan–Meier curve. The prognostic factors of breast carcinoma were assessed on the basis of Cox regression analysis. A nomogram was used to illustrate the prognostic prediction model, and a scoring tool was provided to assess the risk probability. In this study, a nomogram and a calibration plot were drawn with the use of the R package “rms” (Eng et al., 2015) to show the consistency of the actual probability and the predicted risk probability, so as to assess the efficacy of the prognostic model.

### Statistical analysis

Wilcoxon rank-sum test and matched samples *t*-test were used to analyze the expression of *SYCP2* in non-paired samples and paired samples, respectively. Chi-square test or Fisher’s exact test were used to compare and analyze the statistical significance between the two groups of categorical variables, specifically the expression differences of *SYCP2* in different clinicopathological subgroups. Survival curves were drawn using the Kaplan–Meier method, and the differences between groups were assessed *via* the Cox regression analysis. Univariate and multivariate analyses using Cox proportional hazard modeling were performed to estimate the risk of death. Only *p* < 0.05 (bilateral) was considered statistically significant. All statistical analyses and plots were conducted using R (Version 3.6.3).

## Results

### Elevated synaptonemal complex protein-2 expression in breast carcinoma

**FIGURE 2:**
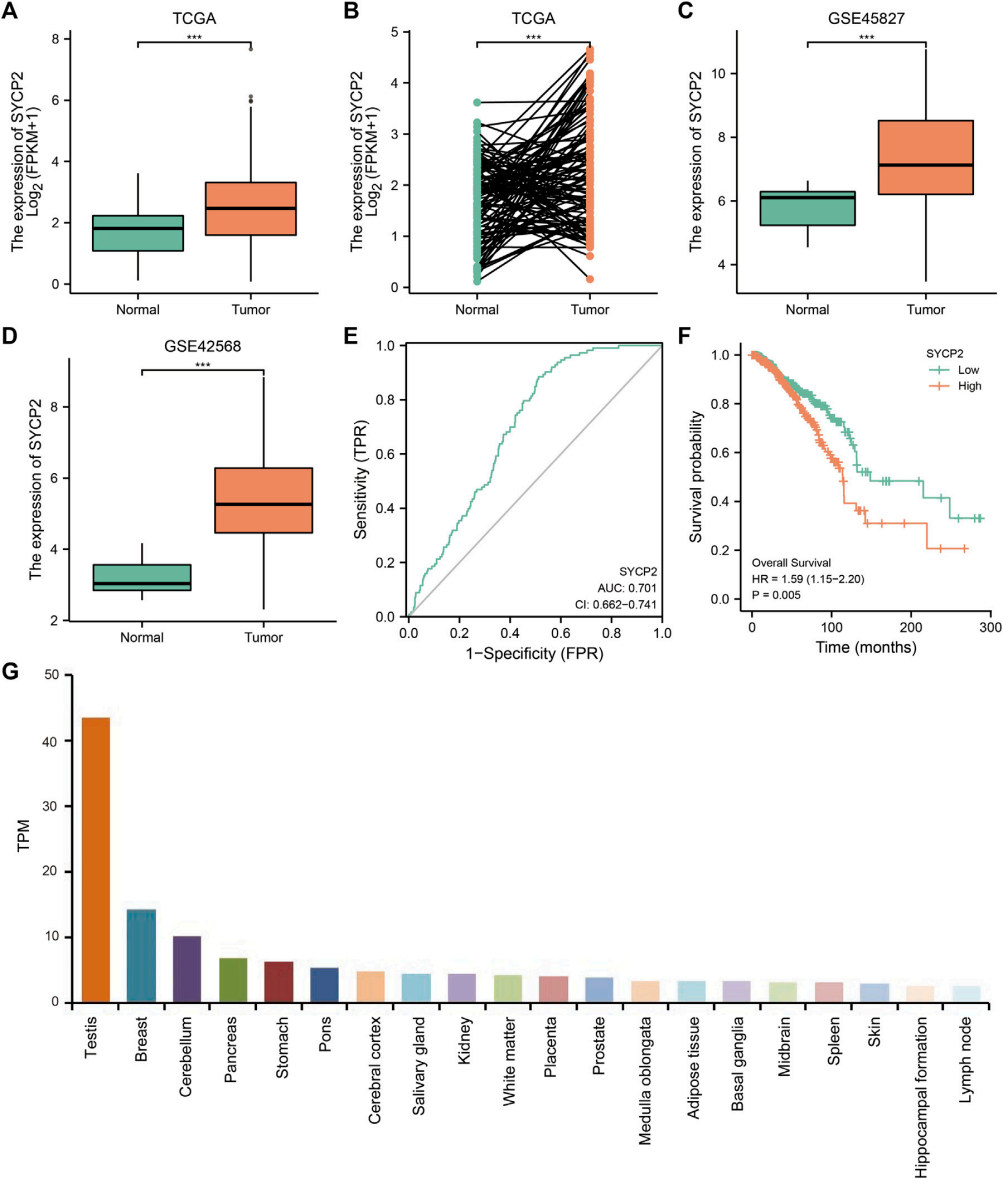
The mRNA and protein expression of *SYCP2* in breast carcinoma. **(A)** The mRNA expression levels of *SYCP2* within breast carcinoma and unpaired-adjacent tissues. **(B)** The mRNA expression levels of *SYCP2* within 112 breast carcinoma and paired-adjacent tissues. **(C)** The mRNA expression levels of *SYCP2* in the GSE45827 dataset. **(D)** The mRNA expression levels of *SYCP2* in the GSE42568 dataset. **(E)** ROC curve revealed the efficiency of the *SYCP2* expression level in distinguishing breast carcinoma tissue from nontumor tissue. **(F)** Kaplan–Meier survival curve comparing the high and low expression of *SYCP2* in breast carcinoma. **(G)** Top 20 distribution of *SYCP2* in systemic tissue expression (ns, *p* ≥ 0.05; *, *p* < 0.05; **, *p* < 0.01; ***, *p* < 0.001).

As depicted in Figure 2, in TCGA-BRCA, GSE45827, and GSE42568, the mRNA expression levels of *SYCP2* in breast carcinoma tissues were significantly higher than that in normal tissues (*p* < 0.001). The ROC curve analysis showed *SYCP2* had an AUC value of 0.701, suggesting that *SYCP2* could be exploited as a potential biomarker. The Kaplan–Meier survival curve indicated that patients suffering from breast carcinoma with high-*SYCP2* mRNA expression had poor prognosis than those with low level of *SYCP2* (*p* = 0.005). In addition, the top 20 tissues with high-*SYCP2* expression are shown in Figure 2.

### Functional and pathway enrichment analyses

**FIGURE 3:**
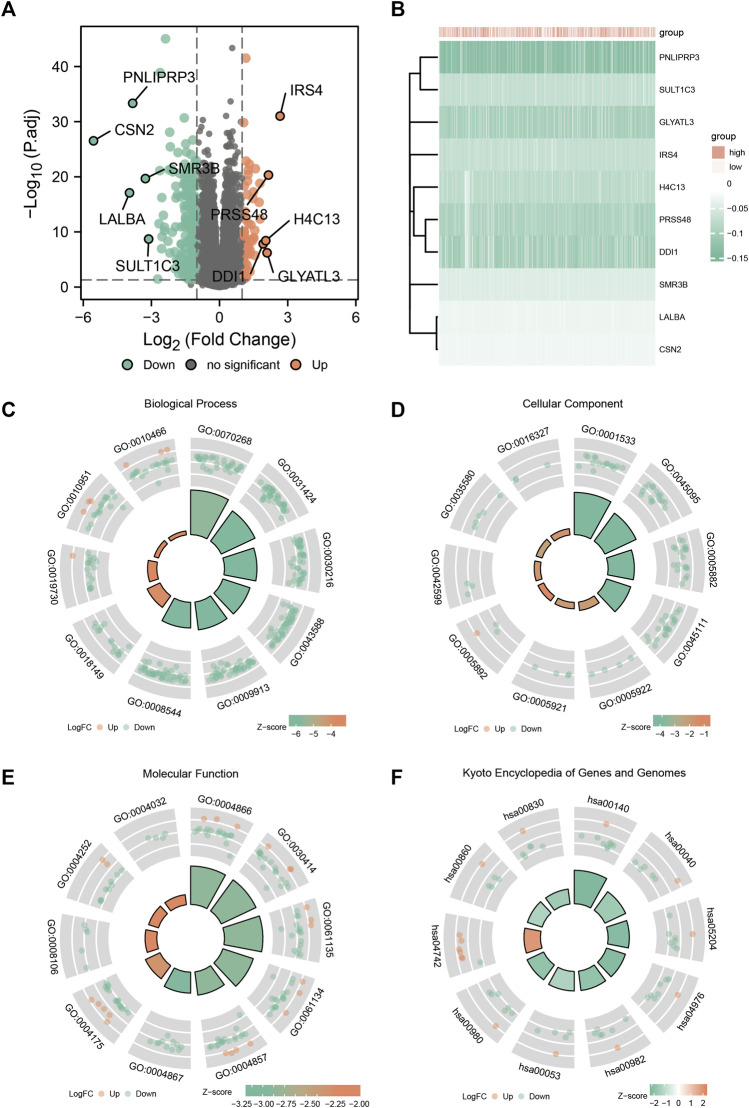
Enrichment analysis of GO and KEGG. **(A)** Volcanic plot of differentially expressed genes in the high-*SYCP2* expression group and the low-*SYCP2* expression group. **(B)** Heatmaps of the top five differentially expressed genes in high- and low-*SYCP2* expression groups. **(C)** Biological process, BP. **(D)** Cellular component, CC. **(E)** Molecular function, MF. **(F)** Kyoto Encyclopedia of Genes and Genomes, KEGG.

**FIGURE 4:**
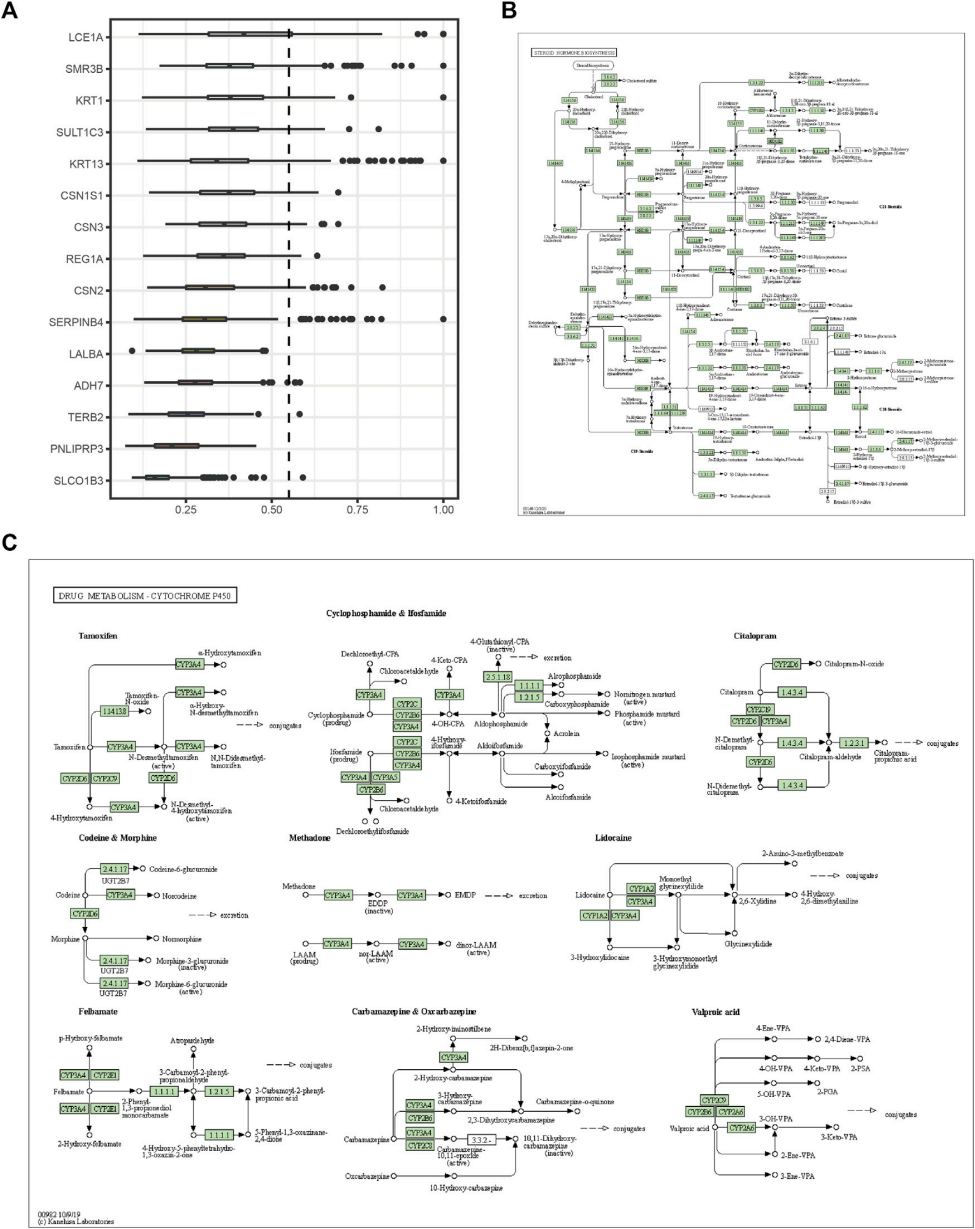
Friends analysis and signaling pathway diagram. **(A)** The top 15 genes that interact with other genes in the pathway from the Friends analysis. **(B)** Steroid hormone biosynthesis. **(C)** Drug metabolism-cytochrome P450.

The expression profiles of the high- and low-*SYCP2* expression groups were compared for identifying DEGs. Moreover, 181 DEGs were obtained, including 244 downregulated genes and 63 upregulated genes (Figure 3A). The heat map showed the expression of the top 5 upregulated and downregulated differential genes between the high- and low-*SYCP2* expression groups (Figure 3B). The DEGs were assigned to three functional groups, including BP, MF, and CC (Table 1). For the BP term, the aforementioned genes showed enrichment in cornification, keratinization, keratinocyte differentiation, skin development, and epidermal cell differentiation (Figure 3C). The CC terms for the above genes were cornified envelope, keratin filament, intermediate filament, intermediate filament cytoskeleton, and intermediate filament cytoskeleton (Figure 3D). The MF terms for the aforementioned genes largely consisted of endopeptidase inhibitor activity, peptidase inhibitor activity, endopeptidase regulator activity, peptidase regulator activity, and enzyme inhibitor activity (Figure 3E). Based on the results of GO analysis, Friend analysis further revealed the top 15 genes that interacted with other genes in the pathway (Figure 4A). The KEGG pathway was enriched in steroid hormone biosynthesis, drug metabolism-cytochrome P450, pentose and glucuronate interconversions, chemical carcinogenesis, bile secretion, ascorbate and aldarate metabolism, and metabolism of xenobiotics by cytochrome P450 (Figures 3F, 4B,C; Table 2).

**TABLE 1:** GO enrichment analysis.

Ontology	ID	Description	Gene ratio	Bg ratio	*p*-value
BP	GO:0070268	Cornification	31/265	112/18,670	1.32e-31
BP	GO:0031424	Keratinization	36/265	224/18,670	1.48e-27
BP	GO:0030216	Keratinocyte differentiation	38/265	305/18,670	6.80e-25
BP	GO:0043588	Skin development	41/265	419/18,670	9.77e-23
BP	GO:0009913	Epidermal cell differentiation	38/265	358/18,670	2.29e-22
BP	GO:0008544	Epidermis development	41/265	464/18,670	4.56e-21
BP	GO:0018149	Peptide crosslinking	15/265	60/18,670	3.87e-15
BP	GO:0019730	Antimicrobial humoral response	17/265	122/18,670	1.56e-12
BP	GO:0010951	Negative regulation of endopeptidase activity	20/265	250/18,670	5.02e-10
BP	GO:0010466	Negative regulation of peptidase activity	20/265	262/18,670	1.14e-09
CC	GO:0001533	Cornified envelope	16/280	65/19,717	6.19e-16
CC	GO:0045095	Keratin filament	16/280	95/19,717	3.60e-13
CC	GO:0005882	Intermediate filament	19/280	214/19,717	2.44e-10
CC	GO:0045111	Intermediate filament cytoskeleton	20/280	251/19,717	5.58e-10
CC	GO:0005922	Connexin complex	4/280	21/19,717	1.97e-04
CC	GO:0005921	Gap junction	4/280	32/19,717	0.001
CC	GO:0005892	Acetylcholine-gated channel complex	3/280	17/19,717	0.002
CC	GO:0042599	Lamellar body	3/280	17/19,717	0.002
CC	GO:0035580	Specific granule lumen	5/280	62/19,717	0.002
CC	GO:0016327	Apicolateral plasma membrane	3/280	18/19,717	0.002
MF	GO:0004866	Endopeptidase inhibitor activity	19/257	175/17,697	1.01e-11
MF	GO:0030414	Peptidase inhibitor activity	19/257	182/17,697	2.03e-11
MF	GO:0061135	Endopeptidase regulator activity	19/257	182/17,697	2.03e-11
MF	GO:0061134	Peptidase regulator activity	19/257	219/17,697	5.02e-10
MF	GO:0004857	Enzyme inhibitor activity	22/257	375/17,697	3.18e-08
MF	GO:0004867	Serine-type endopeptidase inhibitor activity	11/257	94/17,697	1.17e-07
MF	GO:0004175	Endopeptidase activity	21/257	427/17,697	1.22e-06
MF	GO:0008106	Alcohol dehydrogenase (NADP+) activity	5/257	21/17,697	1.05e-05
MF	GO:0004252	Serine-type endopeptidase activity	11/257	160/17,697	2.26e-05
MF	GO:0004032	Alditol:NADP+ 1-oxidoreductase activity	4/257	13/17,697	2.80e-05

**TABLE 2:** KEGG enrichment analysis.

Ontology	ID	Description	Gene ratio	Bg ratio	*p*-value
KEGG	hsa00140	Steroid hormone biosynthesis	9/109	61/8,076	1.03e-07
KEGG	hsa05204	Chemical carcinogenesis	8/109	82/8,076	1.32e-05
KEGG	hsa04976	Bile secretion	8/109	90/8,076	2.63e-05
KEGG	hsa00982	Drug metabolism-cytochrome P450	7/109	71/8,076	4.39e-05
KEGG	hsa00053	Ascorbate and aldarate metabolism	5/109	30/8,076	4.45e-05
KEGG	hsa00980	Metabolism of xenobiotics by cytochrome P450	7/109	77/8,076	7.43e-05
KEGG	hsa04742	Taste transduction	7/109	86/8,076	1.50e-04
KEGG	hsa00860	Porphyrin and chlorophyll metabolism	5/109	42/8,076	2.33e-04
KEGG	hsa00830	Retinol metabolism	6/109	68/8,076	2.92e-04

**FIGURE 5:**
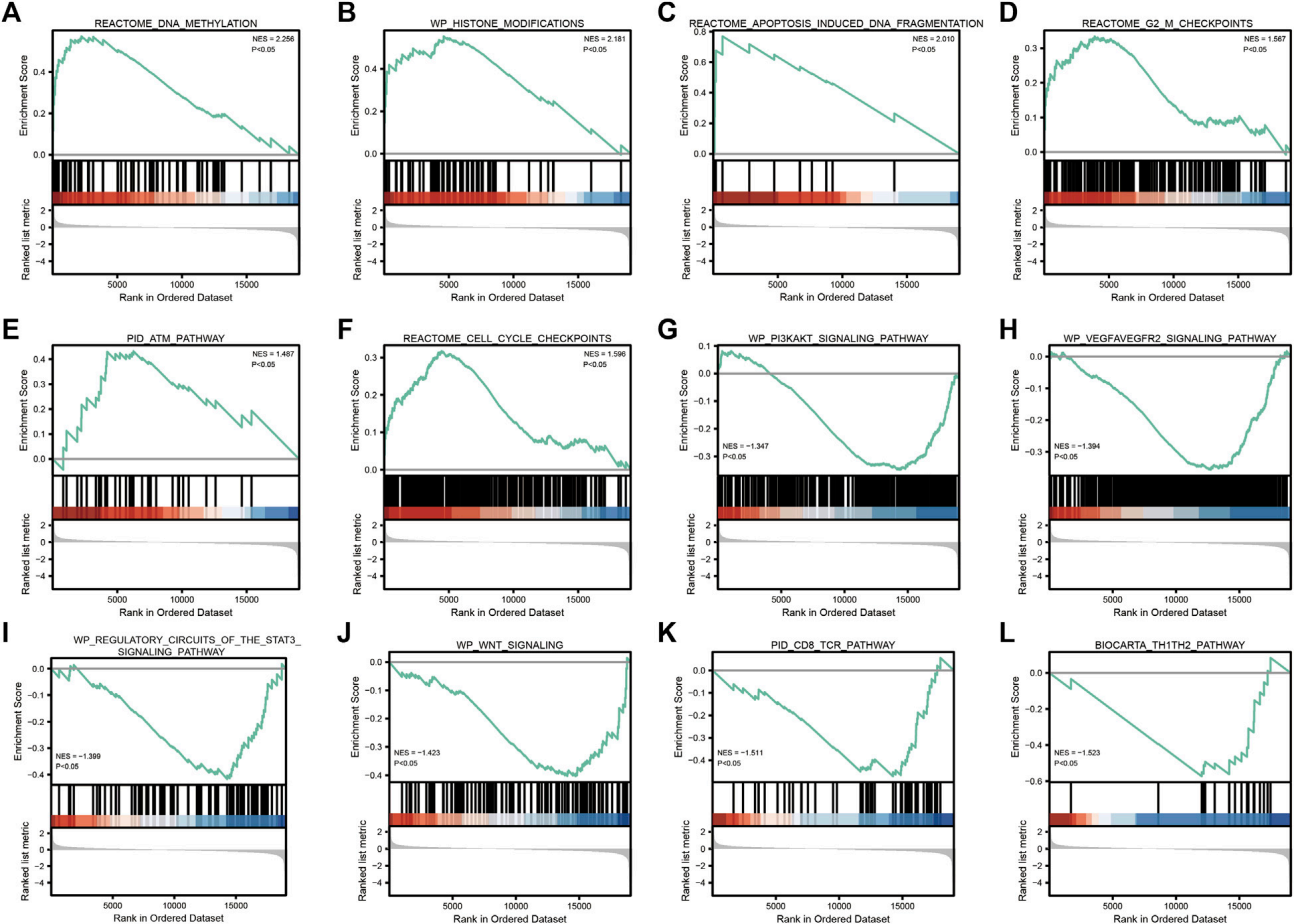
GSEA enrichment analysis. **(A)**: REACTOME_DNA_METHYLATION; **(B)**: WP_HISTONE_MODIFICATIONS; **(C)**: REACTOME_APOPTOSIS_INDUCED_DNA_FRAGMENTATION; **(D)**: REACTOME_G2_M_CHECKPOINTS; **(E)**: PID_ATM_PATHWAY; **(F)**: REACTOME_CELL_CYCLE_CHECKPOINTS; **(G)**: WP_PI3KAKT_SIGNALING_PATHWAY; **(H)**: 、WP_VEGFAVEGFR2_SIGNALING_PATHWAY; **(I)**: WP_REGULATORY_CIRCUITS_OF_THE_STAT3_SIGNALING_PATHWAY; **(J)**: WP_WNT_SIGNALING; **(K)**: PID_CD8_TCR_PATHWAY; **(L)**: BIOCARTA_TH1TH2_PATHWAY”.

GSEA enrichment analysis showed that (Table 3; Figure 5) reactome DNA methylation, wp histone modifications, reactome apoptosis–induced DNA fragmentation, and reactome G2 M checkpoints pathway were significantly enriched in the high-*SYCP2* expression group. The low-*SYCP2* expression group was closely related to the wp PI3KAKT signaling pathway, wp VEGFAVEGFR2 signaling pathway, and wp regulatory circuits of the STAT3 signaling pathway.

**TABLE 3:** GSEA enrichment analysis.

Description	Set size	Enrichment score	NES	*p*-value
REACTOME_DNA_METHYLATION	63	0.571	2.256	0.005
WP_HISTONE_MODIFICATIONS	65	0.554	2.181	0.005
REACTOME_APOPTOSIS_INDUCED_DNA_FRAGMENTATION	13	0.769	2.010	0.003
REACTOME_G2_M_CHECKPOINTS	168	0.334	1.567	0.012
PID_ATM_PATHWAY	34	0.432	1.487	0.048
REACTOME_CELL_CYCLE_CHECKPOINTS	292	0.317	1.596	0.022
WP_PI3KAKT_SIGNALING_PATHWAY	339	−0.348	−1.347	0.013
WP_VEGFAVEGFR2_SIGNALING_PATHWAY	429	−0.356	−1.394	0.003
WP_REGULATORY_CIRCUITS_OF_THE_STAT3_SIGNALING_PATHWAY	78	−0.417	−1.399	0.049
WP_WNT_SIGNALING	113	−0.404	−1.423	0.030
PID_CD8_TCR_PATHWAY	52	−0.471	−1.511	0.029
BIOCARTA_TH1TH2_PATHWAY	21	−0.573	−1.523	0.029

**FIGURE 6:**
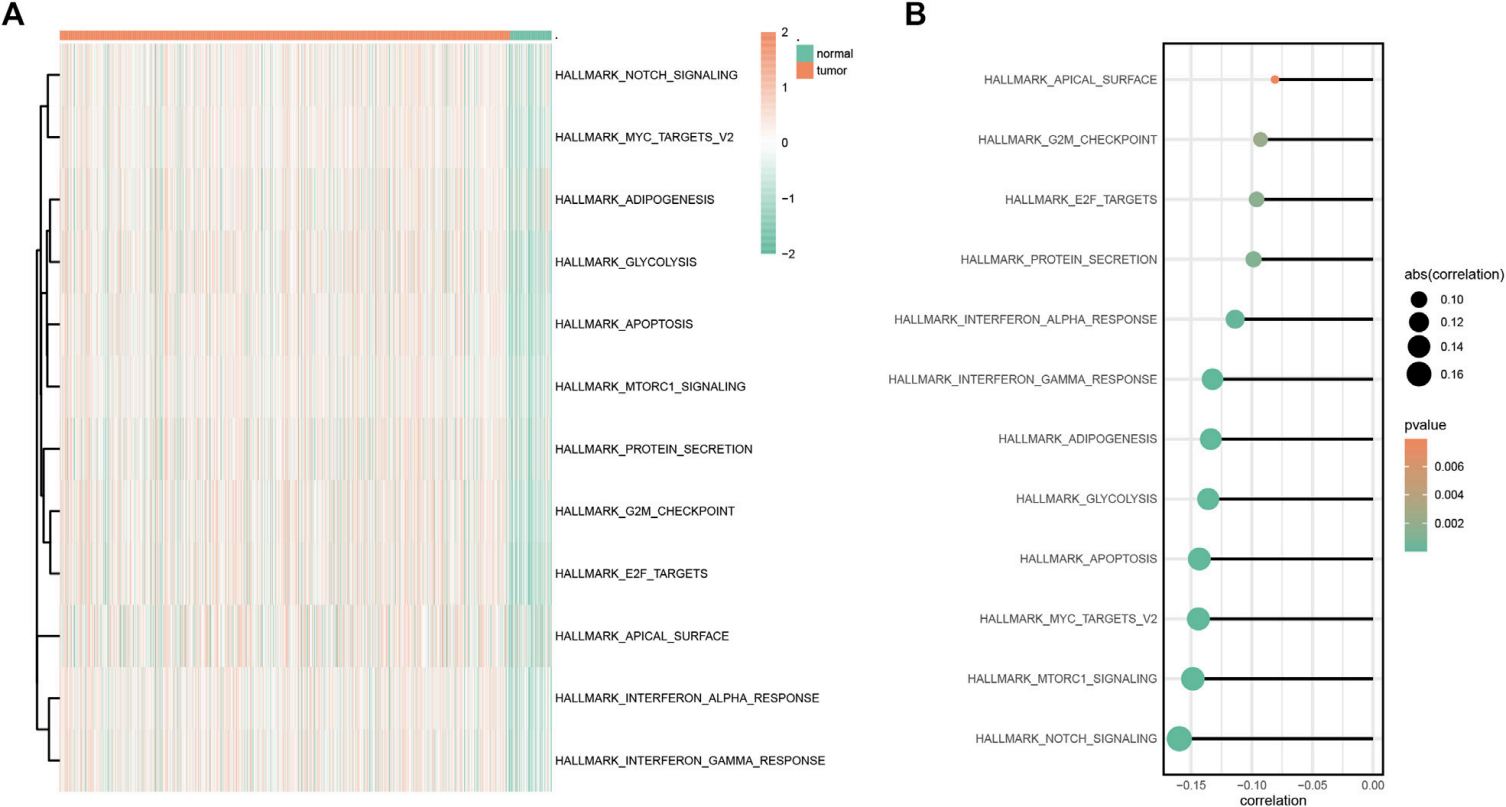
GSVA analysis. **(A)** Heatmap of GSVA differential pathway results based on the hallmark gene set. **(B)** Correlation analysis between differential pathway and the *SYCP2* expression level.

According to GSVA results, multiple hallmark-related pathways differed between tumors and normal tissues (e.g., apoptosis, glycolysis, and notch signaling), and tumor tissues had higher pathway scores than normal tissues (Figure 6A). The higher the score, the greater the difference between the tumor and the normal group. Furthermore, based on the mentioned pathway scores and the *SYCP2* gene expression levels, the correlation analysis suggested that the *SYCP2* expression levels had a negative relationship to the aforementioned pathway scores, that is, the low expression of *SYCP2* was more closely related to these pathways (Figure 6B).

### Construction of interaction network

**FIGURE 7:**
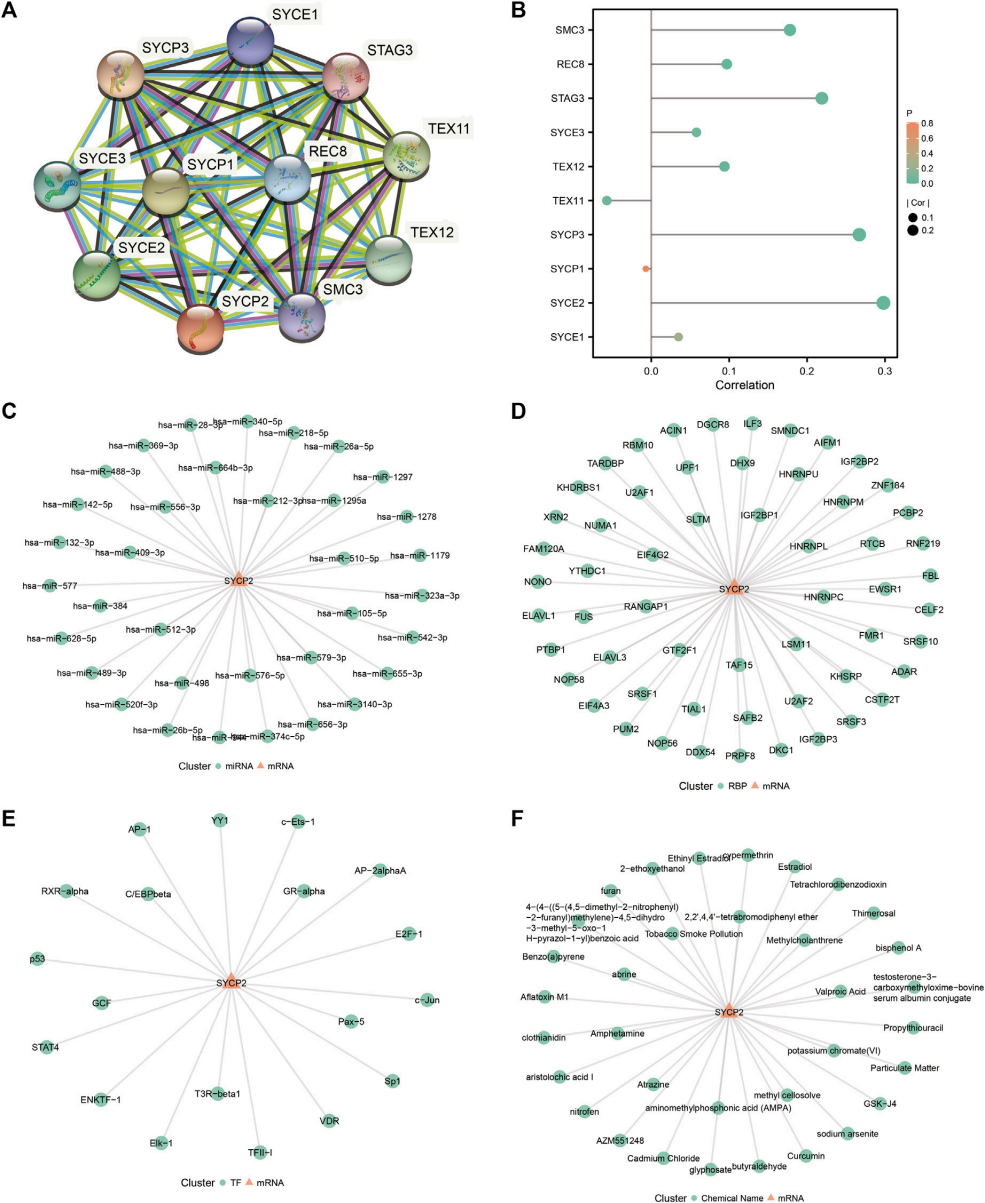
Construction of protein–protein interaction network and related regulatory network. **(A)** PPI network of *SYCP2* based on the STRING database. **(B)** Lollipop diagram of the relationship of *SYCP2* interaction proteins. **(C)** The mRNA–miRNA network was constructed based on the starBase database. **(D)** The mRNA–RBP network was constructed based on the starBase database. **(E)** *SYCP2*-transcription factor network was constructed based on the PROMO platform. **(F)** *SYCP2*-chemical drug network based on CDC database.

The STRING database was used to establish the PPI network of *SYCP2* (Figure 7A), and the lollipop diagram of *SYCP2* interaction proteins was drawn (Figure 7B). The results showed that *SYCP2* had a positive relationship with SYCE2, SYCP3, TEX12, STAG3, REC8, and SMC3 (*p* < 0.05). SYCE2 is part of the synaptonemal complex formed between homologous chromosomes during the meiotic prophase. Similar to *SYCP2*, *SYCP3* encodes an important structural component of the synaptic complex, which is involved in synapsis, recombination, and segregation of meiotic chromosomes. Figures 7C,D present the interaction network constructed by predicting miRNA and RBP based on the starBase database, respectively. Figure 7E illustrates the interaction network based on the transcription factors binding *SYCP2* predicted by the PROMO platform. Chemical drugs associated with the *SYCP2* gene were predicted based on the CDC database (Figure 7F).

### Relationship of synaptonemal complex protein-2 expression and immune infiltration

**FIGURE 8:**
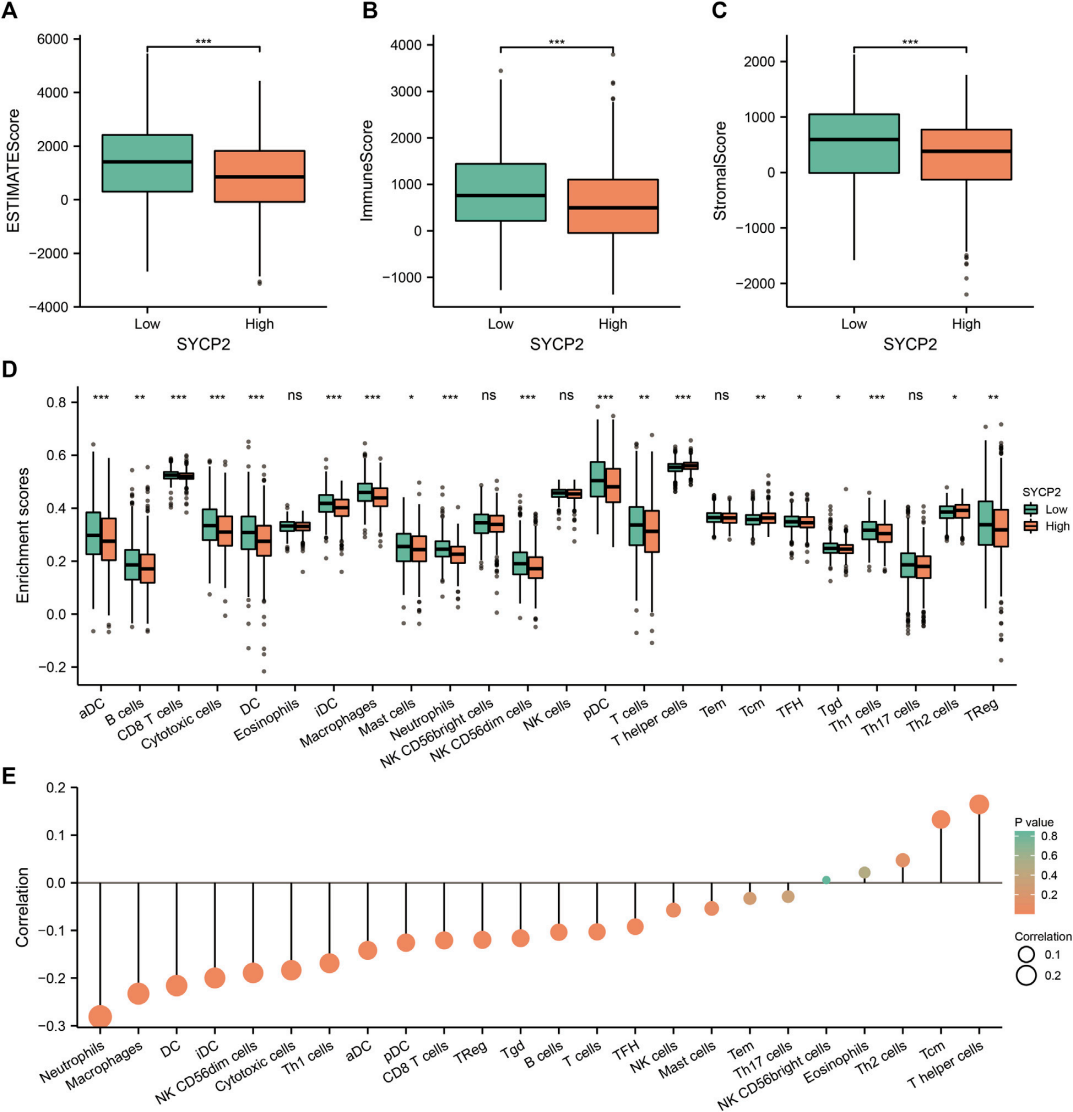
Effects of *SYCP2* gene expression on immunological characteristics of TCGA-BRCA patients. **(A–C)** Compared with patients with low expression of *SYCP2*, patients with high expression of *SYCP2* had a significantly lower ESTIMATE score (*p* < 0.001; Figure 8A), immune score (*p* < 0.001; Figure 8B), and stromal score (*p* < 0.001; Figure 8C). **(D)** The abundance of immune cells in high and low expression samples of *SYCP2* was assessed based on ssGSEA. **(E)** Lollipop diagram of *SYCP2* expression and immune cell infiltration (ns, *p* ≥ 0.05; *, *p* < 0.05; **, *p* < 0.01; ***, *p* < 0.001).

We further analyzed the influence of the expression level of *SYCP2* on the immunological characteristics of TCGA-BRCA patients. Results showed that patients with high-*SYCP2* expression had a significantly lower ESTIMATE score (*p* < 0.001, Figure 8A), immune score (*p* < 0.001, Figure 8B), and stromal score (*p* < 0.001, Figure 8C) compared with patients with low expression of *SYCP2*, which means that there were more tumor cells, immune cells, and stromal cells in the low-*SYCP2* expression group than those in high-*SYCP2* expression samples. In addition, different levels of immune cell infiltration in the high-*SYCP2* expression group and the low-*SYCP2* expression group were analyzed based on ssGSEA, and the result showed that the infiltration levels of aDC, B cells, CD8 T cells, cytotoxic cells, DC, iDC, macrophages, mast cells, neutrophils, NK CD56 dim cells, pDC, T cells, T-helper cells, Tcm, Tfh, Tgd, Th1 cells, Th2 cells, and Treg were significantly different in the high-*SYCP2* expression group and the low-*SYCP2* expression group (*p* < 0.05, Figure 8D). The expression level of *SYCP2* had a positive relationship with the infiltration levels of T-helper cells and Tcm (*p* < 0.001), while having a negative relationship with the infiltration levels of aDC, B cells, CD8 T cells, cytotoxic cells, DC, iDC, macrophages, neutrophils, NK CD56 dim cells, pDC, T cells, Tfh, Tgd, Th1 cells, and Treg (*p* < 0.001, Figure 8E).

### Correlation and prognosis analysis between synaptonemal complex protein-2 expression and clinicopathological characteristics

**FIGURE 9:**
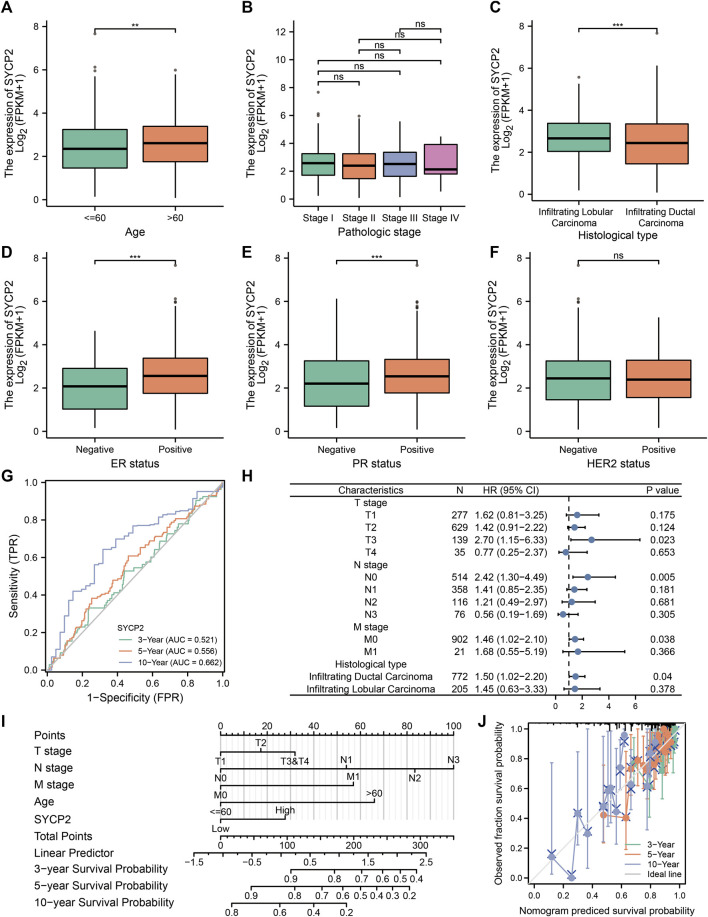
Analysis of clinical characteristics and construction of the prognostic model of patients with the expression profile of *SYCP2* in TCGA-BRCA. **(A,C,D,E)** The expression profile of *SYCP2* showed significant differences in age, pathological type, ER expression, and PR expression (*p* < 0.001). **(B,F)** The expression profile of *SYCP2* showed no significant difference in pathological stage and HER2 expression (*p* > 0.05). **(G)** Time-dependent ROC curve. **(H)** Prognostic forest plot of *SYCP2* in different subgroups. **(I)** Construction of nomogram. **(J)** Calibration plot.

The clinical characteristics of patients with high- and low-*SYCP2* expression in TCGA-BRCA are shown in Table 4 (Supplementary Data Sheet S7). The level of *SYCP2* expression had a significant relationship with age (Figure 9A), histological type (Figure 9C), ER expression (Figure 9D), and PR expression (Figure 9E) (*p* < 0.001). Time-dependent ROC results (Figure 9G) suggested that *SYCP2* was more accurate in predicting prognosis over time. Univariate and multivariate Cox regression analyses showed that age (*p* < 0.001) and the *SYCP2* expression (*p* = 0.049) were independent prognostic factors for TCGA-BRCA patients (Table 5, Supplementary Data Sheet S11). In addition, we also analyzed the prognostic effects of *SYCP2* in different subgroups, and the results showed that *SYCP2* was a risk factor in the subgroup of T3 stage, N0, M0, and infiltrating ductal carcinoma (HR > 1 and *p* < 0.05) (Figure 9H). Subsequently, we constructed a prognostic model based on the above clinical features and drew a nomogram to assess the risk probability (Figure 9I). In addition, the calibration plot indicated that the model has a relatively good predictive value for patients at 3, 5, and 10 years (Figure 9J).

**TABLE 4:** Clinicopathologic characteristics of patients suffering from breast carcinoma with differential *SYCP2* expression.

Characteristic	Levels	Low expression of *SYCP2*	High expression of *SYCP2*	*p*-value
n		541	542	
T stage, n (%)	T1	131 (12.1%)	146 (13.5%)	0.509
	T2	317 (29.4%)	312 (28.9%)	
	T3	71 (6.6%)	68 (6.3%)	
	T4	21 (1.9%)	14 (1.3%)	
N stage, n (%)	N0	257 (24.2%)	257 (24.2%)	0.256
	N1	186 (17.5%)	172 (16.2%)	
	N2	60 (5.6%)	56 (5.3%)	
	N3	30 (2.8%)	46 (4.3%)	
M stage, n (%)	M0	448 (48.6%)	454 (49.2%)	0.806
	M1	11 (1.2%)	9 (1%)	
Pathologic stage, n (%)	Stage I	85 (8%)	96 (9.1%)	0.570
	Stage II	320 (30.2%)	299 (28.2%)	
	Stage III	116 (10.9%)	126 (11.9%)	
	Stage IV	10 (0.9%)	8 (0.8%)	
Age, n (%)	≤60	325 (30%)	276 (25.5%)	0.003
	>60	216 (19.9%)	266 (24.6%)	
Histological type, n (%)	Infiltrating ductal carcinoma	394 (40.3%)	378 (38.7%)	0.009
	Infiltrating lobular Carcinoma	83 (8.5%)	122 (12.5%)	
PR status, n (%)	Negative	189 (18.3%)	153 (14.8%)	0.006
	Indeterminate	4 (0.4%)	0 (0%)	
	Positive	328 (31.7%)	360 (34.8%)	
ER status, n (%)	Negative	146 (14.1%)	94 (9.1%)	<0.001
	Indeterminate	1 (0.1%)	1 (0.1%)	
	Positive	374 (36.1%)	419 (40.5%)	
HER2 status, n (%)	Negative	283 (38.9%)	275 (37.8%)	0.802
	Indeterminate	5 (0.7%)	7 (1%)	
	Positive	81 (11.1%)	76 (10.5%)	
Age, median (IQR)		56 (47, 66)	60 (50, 68)	<0.001

**TABLE 5:** Univariate and multivariate Cox expression analysis.

Characteristics	Total (N)	Univariate analysis		Multivariate analysis	
		Hazard ratio (95% CI)	*p*-value	Hazard ratio (95% CI)	*p*-value
T stage	1,079				
T1	276	References			
T2	629	1.332 (0.887–1.999)	0.166	1.117 (0.452–2.758)	0.810
T3 and T4	174	1.953 (1.221–3.123)	**0.005**	2.590 (0.852–7.875)	0.093
N stage	1,063				
N0	514	References			
N1	357	1.956 (1.329–2.879)	**<0.001**	1.448 (0.675–3.108)	0.342
N2	116	2.519 (1.482–4.281)	**<0.001**	1.278 (0.355–4.609)	0.707
N3	76	4.188 (2.316–7.574)	**<0.001**	2.960 (0.818–10.711)	0.098
M stage	922				
M0	902	References			
M1	20	4.254 (2.468–7.334)	**<0.001**	3.211 (0.509–20.234)	0.214
Pathologic stage	1,059				
Stage I	180	References			
Stage II	619	1.697 (0.985–2.922)	0.057	0.811 (0.244–2.695)	0.732
Stage III	242	2.962 (1.664–5.273)	**<0.001**	1.486 (0.251–8.795)	0.662
Stage IV	18	11.607 (5.569–24.190)	**<0.001**		
Race	993				
Asian	60	References			
Black or African American	180	1.525 (0.463–5.024)	0.488		
White	753	1.325 (0.420–4.186)	0.631		
Histological type	977				
Infiltrating ductal carcinoma	772	References			
Infiltrating lobular carcinoma	205	0.827 (0.526–1.299)	0.410		
PR status	1,029				
Negative	342	References			
Positive	687	0.732 (0.523–1.024)	0.068	0.931 (0.425–2.039)	0.858
ER status	1,032				
Negative	240	References			
Positive	792	0.712 (0.495–1.023)	0.066	0.444 (0.194–1.013)	0.054
HER2 status	715				
Negative	558	References			
Positive	157	1.593 (0.973–2.609)	0.064	1.028 (0.576–1.834)	0.926
Age	1,082				
≤60	601	References			
>60	481	2.020 (1.465–2.784)	**<0.001**	3.142 (1.885–5.238)	**<0.001**
*SYCP2*	1,082				
Low	540	References			
High	542	1.594 (1.152–2.204)	**0.005**	1.653 (1.002–2.725)	**0.049**

The bold values indicate that the results are statistically significant.

## Discussion

*SYCP2* is the most crucial gene in terms of chromosomal synapsis and synaptonemal complex assembly in the course of male meiosis (Yang et al., 2006). Researchers identified exclusive overexpression of *SYCP2* from the der (20) allele and revealed three heterozygous *SYCP2* frameshift variants in other subjects with cryptozoospermia and azoospermia according to exome sequencing of infertile males (Schilit et al., 2020). SC is a specific structure formed by homologous chromosomes during the prophase of meiosis, which promotes double-strand break (DSB) formation (Hollingsworth, 2020). Therefore, the encoded protein of *SYCP2* is involved in the cell cycle, specifically in the M phase (mitosis and cytokinesis) (Espinosa et al., 2013). While *SYCP2* is a testicular-specific human gene, elevated *SYCP2* expression is likely to result in the genomic instability arising from high-risk HPV infection and the following oncogenic change (Masterson et al., 2015) in HPV-related carcinomas, which consist of cervical squamous cell carcinoma (CSCC) (Li et al., 2021; Luo et al., 2021) as well as head and neck squamous cell carcinoma (HNSCC) (Tripathi et al., 2020; Mendez-Matias et al., 2021; Berglund et al., 2022). As indicated by existing studies, *SYCP2* belonging to the mitosis pathway is likely to play a certain role in the oncogenesis of cervical carcinoma and can be used as a diagnostic marker and therapeutic target (Espinosa et al., 2013); *SYCP2* with alternative spliced events is likely to facilitate the CSCC progression (Guo et al., 2015). However, *SYCP2* expression remains unclear, and its prognostic value in breast carcinoma has not been confirmed. In this study, bioinformatics analysis was conducted on the expression levels and prognostic value of *SYCP2* in breast carcinoma with the use of high-throughput transcriptomic data that originated from TCGA/GEO. It was confirmed that *SYCP2* is significantly upregulated in breast carcinoma tissues as compared with normal samples, and patients with high-*SYCP2* expression had a poor prognosis than those with low-*SYCP2* expression. Moreover, the ROC curves suggested the significant diagnostic ability of *SYCP2* for breast carcinoma. The aforementioned data indicated that *SYCP2* might serve as a potential prognostic marker in breast carcinoma.

GO, KEGG, GSEA, and GSVA were performed to investigate the underlying functions and mechanisms of *SYCP2* in breast carcinoma in depth. GSEA revealed that DNA methylation, histone modification, apoptosis-induced DNA fragmentation, and G2/M checkpoints are differentially enriched in the high-*SYCP2* expression group. As reported by a recent study, the aberrant expression of *SYCP2* was related to the methylation status of multiple CpG sites in both luminal A and luminal B patients. Moreover, researchers suggested that HPV infection in HNSCC is associated with type-specific methylomic profiles, and *SYCP2* is one of the significant differentially methylated genes (Berglund et al., 2022). Friends analysis based on the GO analysis results found hub genes in the pathway, which consisted of numerous methylation-related genes, especially CSN1S1. Lower expression of CSN1S1 could be monitored due to promoter methylation, mutations, and copy number alteration (CNA) (Mou et al., 2020). The aforementioned findings provided evidence that DNA methylation, especially HPV type-specific methylomic changes, significantly affects the development and progression of breast carcinoma. In addition, for the BP and CC in the results of this study of GO analysis, the DEGs were enriched in cornification, keratinization, keratinocyte differentiation, cornified envelope, and keratin filament. Keratins refer to epithelium-specific intermediate filament proteins that take on a critical significance for enhancing the structural integrity and polarity of cells and are considered to be involved in cell differentiation (Desai et al., 2009; Green et al., 2019). Under normal physiological conditions, keratinocytes are consistent with a specific process of apoptotic cell death and terminal differentiation, thus ultimately resulting in the formation of the keratin layer (Eckhart et al., 2013). Some studies have suggested that some decreased expression of keratins contributes to an initiation of metastasis by loosening cell adhesion through disassembly of desmosomes during distinct epithelial–mesenchymal transition (EMT) states (Huang et al., 2012; Seltmann et al., 2013). Accordingly, it has been suggested that the role of keratin in maintaining intercellular adhesion can act as a protective barrier against EMT and cell migration (Kroger et al., 2013). However, an upregulated expression of certain keratins has been found to facilitate cell migration and invasion in multiple malignancies (Cheung et al., 2013; Chung et al., 2015), and the possible mechanism involved in the invasion of extracellular matrix collectively by tumor cells (Cheung and Ewald, 2014; Yang et al., 2019). Thus, further investigations are warranted to elucidate the direct molecular mechanisms of the underlying interactions between *SYCP2* expression and keratinocyte differentiation. Meanwhile, increasing evidence suggested that the viral oncoproteins can facilitate keratinocyte immortalization and disrupt the normal cytokeratin (CK) expression pattern in stratified squamous epithelium (Sun et al., 1993), by the stepwise process that leads to the oncogenesis of squamous cell carcinoma. Thus, we speculated whether patients suffering from breast carcinoma are also accompanied by HPV infection, and whether *SYCP2* might affect the prognosis of patients suffering from breast carcinoma by regulating keratinocyte differentiation, which needs further verification.

The PPI results showed that *SYCP2* had a positive relationship with SYCE2, SYCP3, TEX12, STAG3, REC8, and SMC3. SYCE2, SYCP2, and SYCP3 are all important components of the synaptonemal complex (SC), which refers to a type of meiosis-specific nuclear structure playing a critical role in proper segregation, recombination, and synapsis of homologous chromosomes. STAG3 is a subunit of the cohesin complex which regulates the cohesion of sister chromatids during cell division. REC8 and SMC3 belong to the subfamily of structural maintenance of chromosomes (SMC) proteins which is a component of the multimeric cohesin complex that holds together sister chromatids during mitosis. It appears that these genes have similar or interconnected functional significance. Another potential concern of this study is that *SYCP2* expression is significantly related to multiple immune infiltration levels of TCGA-BRCA patients. First, the ESTIMATE score, immune score, and stromal score in the high-*SYCP2* expression group were significantly lower than those in the low-*SYCP2* expression group, suggesting that *SYCP2* is a crucial immune-related gene. Second, the relationship between *SYCP2* expression and the immune cells implicates the role of *SYCP2* in the regulation tumor immunology in breast carcinoma. To be specific, the expression level of *SYCP2* had a positive relationship with infiltration levels of T-helper cells and Tcm, while having a negative relationship with multiple immune cells (e.g., DC, aDC, pDC, iDC, neutrophils, and macrophages). T lymphocytes play a crucial role in the progression of breast carcinoma, especially in triple-negative breast carcinoma (Zhou et al., 2022). CD4^+^ T-helper cells directly or indirectly exert protumorigenic or/and antitumorigenic immune effects by affecting other immune cells (Criscitiello et al., 2016). Furthermore, anti-PD-1 therapy regulates systemic immune reactions, and exerts antineoplastic effects, not only by revitalizing Tem and Tcm of CD4^+^ and CD8^+^ T cells, but also *via* a shift to a Th1 phenotype (Yamaguchi et al., 2018). DCs are a heterogeneous population of antigen-presenting cells (APCs), containing a variety of subsets, that play critical roles in promoting an immune response against antigens including foreign pathogenic antigens and self-tumor antigens (Balan et al., 2019). Although DCs contribute to a small part of the tumor microenvironment, they are emerging as an essential antitumor component since they can stimulate tumor-specific T-cell responses and immunotherapy responses (de Winde et al., 2020; Sadeghzadeh et al., 2020). Consequently, the results of this study revealed that *SYCP2* has the potential to affect immune cell infiltration and interfere with immunotherapy, providing evidence for its use as a predictive biomarker for immunotherapy in patients with breast carcinoma.

The clinicopathological characteristics of patients with high- and low-*SYCP2* expression suggested that the level of *SYCP2* expression had a significant relationship with pathological type, ER expression, and PR expression, thus suggesting that the high association between *SYCP2* expression level and survival might be affected by histopathological type and differentiation. In stratified analysis, we found that *SYCP2* expression remained a powerful forecaster of the prognosis within the subsets, including the T3 stage, N0, M0, and infiltrating ductal carcinoma. In addition, Cox regression analysis showed that the *SYCP2* could act as an independent prognostic factor of TCGA-BRCA patients. Moreover, as revealed by the *SYCP2*-related nomogram of this study, *SYCP2* made a larger contribution to OS, compared with FIGO stage and histological grade. The calibration plot revealed that the *SYCP2* model has a relatively good predictive value for 3 years, 5 years, and 10 years of survival, and the prediction efficiency of *SYCP2* becomes more accurate as time goes by.

Although the results of this study provided more insights into the relationship between *SYCP2* and breast carcinoma, there are certain limitations that have to be mentioned. First, the relationship between *SYCP2* expression and the OS of overall patients suffering from breast carcinoma was investigated, instead of the relationship between *SYCP2* expression and the OS of patients suffering from each subtype of breast carcinoma. In-depth studies that include larger sample sizes should be conducted for validating the findings of this study and exploring the prognostic value of *SYCP2* in the clinical management of breast carcinoma. Second, the *SYCP2* mRNA and protein expression should be verified through cytological experiments with the use of clinical samples, which are the focus of the next steps. Lastly, the potential mechanisms of distinct *SYCP2* in breast carcinoma were investigated. However, the specific mechanism of *SYCP2* in breast carcinoma remains unclear.

In brief, this study suggested that elevated *SYCP2* expression has a prognostic value for individuals suffering from breast carcinoma and *SYCP2* may act as a potential prognostic molecular marker of poor survival. DNA methylation, keratinocyte differentiation, steroid hormone biosynthesis, and immune infiltration are likely to be the vital pathway regulated by *SYCP2*. Accordingly, this study may provide a reference for the development of prognostic indicators and novel therapeutic targets in patients suffering from breast carcinoma.

## Data Availability

The datasets presented in this study can be found in online repositories. The names of the repository/repositories and accession number(s) can be found in the article/Supplementary Material.

## Glossary

aDC: activated DC
BP: biological processes
CC: cellular components
CSCC: cervical squamous cell carcinoma
DC: dendritic cell
DEGs: differentially expressed genes
DSBs: double-strand breaks
GEO: Gene Expression Omnibus
GO: Gene Ontology
GSEA: gene set enrichment analysis
HNSCC: head and neck squamous cell carcinoma
HPA: Human Protein Atlas
HPV: human papillomavirus
iDC: immature DC
KEGG: Kyoto Encyclopedia of Genes and Genomes
MF: molecular functions
MSigDB: Molecular Signatures Database
OS: overall survival
pDC: plasmacytoid DC
PPI: protein–protein interaction
RBP: RNA-binding proteins
ROC: receiver operating characteristic
SC: synaptonemal complex
ssGSEA: single-sample GSEA
SYCP2: synaptonemal complex protein-2
TCGA: The Cancer Genome Atlas
Tcm: T central memory
Tem: T-effector memory
Tfh: T-follicular helper
Tgd: T-gamma delta
Treg: regulatory T cell

## References

[B1] Balan S. Saxena M. Bhardwaj N. (2019). Dendritic cell subsets and locations. Int. Rev. Cell Mol. Biol. 348, 1–68. 10.1016/bs.ircmb.2019.07.004 31810551

[B2] Barbie D. A. Tamayo P. Boehm J. S. Kim S. Y. Moody S. E. Dunn I. F. (2009). Systematic RNA interference reveals that oncogenic KRAS-driven cancers require TBK1. Nature 462 (7269), 108–112. 10.1038/nature08460 19847166 PMC2783335

[B3] Berglund A. Muenyi C. Siegel E. M. Ajidahun A. Eschrich S. A. Wong D. (2022). Characterization of epigenomic alterations in HPV16+ head and neck squamous cell carcinomas. Cancer Epidemiol. Biomarkers Prev. 1158, 858–869. 10.1158/1055-9965.EPI-21-0922 PMC8983563 35064062

[B4] Bray F. Ferlay J. Soerjomataram I. Siegel R. L. Torre L. A. Jemal A. (2018). Global cancer statistics 2018: GLOBOCAN estimates of incidence and mortality worldwide for 36 cancers in 185 countries. Ca. Cancer J. Clin. 68 (6), 394–424. 10.3322/caac.21492 30207593

[B5] Cheung K. J. Ewald A. J. (2014). Illuminating breast cancer invasion: Diverse roles for cell-cell interactions. Curr. Opin. Cell Biol. 30, 99–111. 10.1016/j.ceb.2014.07.003 25137487 PMC4250974

[B6] Cheung K. J. Gabrielson E. Werb Z. Ewald A. J. (2013). Collective invasion in breast cancer requires a conserved basal epithelial program. Cell 155 (7), 1639–1651. 10.1016/j.cell.2013.11.029 24332913 PMC3941206

[B7] Chung B. M. Arutyunov A. Ilagan E. Yao N. Wills-Karp M. Coulombe P. A. (2015). Regulation of C-X-C chemokine gene expression by keratin 17 and hnRNP K in skin tumor keratinocytes. J. Cell Biol. 208 (5), 613–627. 10.1083/jcb.201408026 25713416 PMC4347647

[B8] Clarke C. Madden S. F. Doolan P. Aherne S. T. Joyce H. O'Driscoll L. (2013). Correlating transcriptional networks to breast cancer survival: A large-scale coexpression analysis. Carcinogenesis 34 (10), 2300–2308. 10.1093/carcin/bgt208 23740839

[B9] Criscitiello C. Esposito A. Trapani D. Curigliano G. (2016). Prognostic and predictive value of tumor infiltrating lymphocytes in early breast cancer. Cancer Treat. Rev. 50, 205–207. 10.1016/j.ctrv.2016.09.019 27744144

[B10] Davis A. P. Grondin C. J. Johnson R. J. Sciaky D. Wiegers J. Wiegers T. C. (2021). Comparative toxicogenomics database (CTD): Update 2021. Nucleic Acids Res. 49 (D1), D1138–D1143. 10.1093/nar/gkaa891 33068428 PMC7779006

[B11] de Winde C. M. Munday C. Acton S. E. (2020). Molecular mechanisms of dendritic cell migration in immunity and cancer. Med. Microbiol. Immunol. 209 (4), 515–529. 10.1007/s00430-020-00680-4 32451606 PMC7395046

[B12] Desai B. V. Harmon R. M. Green K. J. (2009). Desmosomes at a glance. J. Cell Sci. 122 (24), 4401–4407. 10.1242/jcs.037457 19955337 PMC2787455

[B13] Eckhart L. Lippens S. Tschachler E. Declercq W. (2013). Cell death by cornification. Biochim. Biophys. Acta 1833 (12), 3471–3480. 10.1016/j.bbamcr.2013.06.010 23792051

[B14] Eng K. H. Schiller E. Morrell K. (2015). On representing the prognostic value of continuous gene expression biomarkers with the restricted mean survival curve. Oncotarget 6 (34), 36308–36318. 10.18632/oncotarget.6121 26486086 PMC4742179

[B15] Espinosa A. M. Alfaro A. Roman-Basaure E. Guardado-Estrada M. Palma I. Serralde C. (2013). Mitosis is a source of potential markers for screening and survival and therapeutic targets in cervical cancer. PLoS One 8 (2), e55975. 10.1371/journal.pone.0055975 23405241 PMC3566100

[B16] Farre D. Roset R. Huerta M. Adsuara J. E. Rosello L. Alba M. M. (2003). Identification of patterns in biological sequences at the ALGGEN server: PROMO and MALGEN. Nucleic Acids Res. 31 (13), 3651–3653. 10.1093/nar/gkg605 12824386 PMC169011

[B17] Feng J. Fu S. Cao X. Wu H. Lu J. Zeng M. (2017). Synaptonemal complex protein 2 (SYCP2) mediates the association of the centromere with the synaptonemal complex. Protein Cell 8 (7), 538–543. 10.1007/s13238-016-0354-6 28150150 PMC5498334

[B18] Fraune J. Alsheimer M. Redolfi J. Brochier-Armanet C. Benavente R. (2014). Protein SYCP2 is an ancient component of the metazoan synaptonemal complex. Cytogenet. Genome Res. 144 (4), 299–305. 10.1159/000381080 25831978

[B19] Green K. J. Jaiganesh A. Broussard J. A. (2019). Desmosomes: Essential contributors to an integrated intercellular junction network. F1000Res 8, F1000. 10.12688/f1000research.20942.1 PMC6944264 31942240

[B20] Gruosso T. Mieulet V. Cardon M. Bourachot B. Kieffer Y. Devun F. (2016). Chronic oxidative stress promotes H2AX protein degradation and enhances chemosensitivity in breast cancer patients. EMBO Mol. Med. 8 (5), 527–549. 10.15252/emmm.201505891 27006338 PMC5123617

[B21] Guo P. Wang D. Wu J. Yang J. Ren T. Zhu B. (2015). The landscape of alternative splicing in cervical squamous cell carcinoma. Onco. Targets. Ther. 8, 73–79. 10.2147/OTT.S72832 25565867 PMC4278777

[B22] Hanzelmann S. Castelo R. Guinney J. (2013). Gsva: Gene set variation analysis for microarray and RNA-seq data. BMC Bioinforma. 14, 7. 10.1186/1471-2105-14-7 PMC3618321 23323831

[B23] Hollingsworth N. M. (2020). A new role for the synaptonemal complex in the regulation of meiotic recombination. Genes Dev. 34 (23-24), 1562–1564. 10.1101/gad.345488.120 33262143 PMC7706701

[B24] Huang R. Y. Guilford P. Thiery J. P. (2012). Early events in cell adhesion and polarity during epithelial-mesenchymal transition. J. Cell Sci. 125 (19), 4417–4422. 10.1242/jcs.099697 23165231

[B25] Kouznetsova A. Novak I. Jessberger R. Hoog C. (2005). SYCP2 and SYCP3 are required for cohesin core integrity at diplotene but not for centromere cohesion at the first meiotic division. J. Cell Sci. 118 (10), 2271–2278. 10.1242/jcs.02362 15870106

[B26] Kroger C. Loschke F. Schwarz N. Windoffer R. Leube R. E. Magin T. M. (2013). Keratins control intercellular adhesion involving PKC-alpha-mediated desmoplakin phosphorylation. J. Cell Biol. 201 (5), 681–692. 10.1083/jcb.201208162 23690176 PMC3664716

[B27] Li J. H. Liu S. Zhou H. Qu L. H. Yang J. H. (2014). starBase v2.0: decoding miRNA-ceRNA, miRNA-ncRNA and protein-RNA interaction networks from large-scale CLIP-Seq data. Nucleic Acids Res. 42, D92–D97. 10.1093/nar/gkt1248 24297251 PMC3964941

[B28] Li Z. Chen J. Zhao S. Li Y. Zhou J. Liang J. (2021). Discovery and validation of novel biomarkers for detection of cervical cancer. Cancer Med. 10 (6), 2063–2074. 10.1002/cam4.3799 33624385 PMC7957177

[B29] Love M. I. Huber W. Anders S. (2014). Moderated estimation of fold change and dispersion for RNA-seq data with DESeq2. Genome Biol. 15 (12), 550. 10.1186/s13059-014-0550-8 25516281 PMC4302049

[B30] Luo H. Li Y. Zhao Y. Chang J. Zhang X. Zou B. (2021). Comprehensive analysis of circRNA expression profiles during cervical carcinogenesis. Front. Oncol. 11, 676609. 10.3389/fonc.2021.676609 34532284 PMC8438239

[B31] Martinez I. Wang J. Hobson K. F. Ferris R. L. Khan S. A. (2007). Identification of differentially expressed genes in HPV-positive and HPV-negative oropharyngeal squamous cell carcinomas. Eur. J. Cancer 43 (2), 415–432. 10.1016/j.ejca.2006.09.001 17079134 PMC1847595

[B32] Masterson L. Sorgeloos F. Winder D. Lechner M. Marker A. Malhotra S. (2015). Deregulation of SYCP2 predicts early stage human papillomavirus-positive oropharyngeal carcinoma: A prospective whole transcriptome analysis. Cancer Sci. 106 (11), 1568–1575. 10.1111/cas.12809 26334652 PMC4714680

[B33] Mendez-Matias G. Velazquez-Velazquez C. Castro-Oropeza R. Mantilla-Morales A. Ocampo-Sandoval D. Burgos-Gonzalez A. (2021). Prevalence of HPV in Mexican patients with head and neck squamous carcinoma and identification of potential prognostic biomarkers. Cancers (Basel) 13 (22), 5602. 10.3390/cancers13225602 34830760 PMC8616077

[B34] Messeguer X. Escudero R. Farre D. Nunez O. Martinez J. Alba M. M. (2002). Promo: Detection of known transcription regulatory elements using species-tailored searches. Bioinformatics 18 (2), 333–334. 10.1093/bioinformatics/18.2.333 11847087

[B35] Mou M. A. Keya N. A. Islam M. Hossain M. J. Al Habib M. S. Alam R. (2020). Validation of CSN1S1 transcriptional expression, promoter methylation, and prognostic power in breast cancer using independent datasets. Biochem. Biophys. Rep. 24, 100867. 10.1016/j.bbrep.2020.100867 33381666 PMC7767798

[B36] Offenberg H. H. Schalk J. A. Meuwissen R. L. van Aalderen M. Kester H. A. Dietrich A. J. (1998). SCP2: A major protein component of the axial elements of synaptonemal complexes of the rat. Nucleic Acids Res. 26 (11), 2572–2579. 10.1093/nar/26.11.2572 9592139 PMC147596

[B37] Ritchie M. E. Phipson B. Wu D. Hu Y. Law C. W. Shi W. (2015). Limma powers differential expression analyses for RNA-sequencing and microarray studies. Nucleic Acids Res. 43 (7), e47. 10.1093/nar/gkv007 25605792 PMC4402510

[B38] Sadeghzadeh M. Bornehdeli S. Mohahammadrezakhani H. Abolghasemi M. Poursaei E. Asadi M. (2020). Dendritic cell therapy in cancer treatment; the state-of-the-art. Life Sci. 254, 117580. 10.1016/j.lfs.2020.117580 32205087

[B39] Schilit S. L. P. Menon S. Friedrich C. Kammin T. Wilch E. Hanscom C. (2020). SYCP2 translocation-mediated dysregulation and frameshift variants cause human male infertility. Am. J. Hum. Genet. 106 (1), 41–57. 10.1016/j.ajhg.2019.11.013 31866047 PMC7042487

[B40] Seltmann K. Fritsch A. W. Kas J. A. Magin T. M. (2013). Keratins significantly contribute to cell stiffness and impact invasive behavior. Proc. Natl. Acad. Sci. U. S. A. 110 (46), 18507–18512. 10.1073/pnas.1310493110 24167274 PMC3832002

[B41] Subramanian A. Tamayo P. Mootha V. K. Mukherjee S. Ebert B. L. Gillette M. A. (2005). Gene set enrichment analysis: A knowledge-based approach for interpreting genome-wide expression profiles. Proc. Natl. Acad. Sci. U. S. A. 102 (43), 15545–15550. 10.1073/pnas.0506580102 16199517 PMC1239896

[B42] Sun Q. Tsutsumi K. Yokoyama M. Pater M. M. Pater A. (1993). *In vivo* cytokeratin-expression pattern of stratified squamous epithelium from human papillomavirus-type-16-immortalized ectocervical and foreskin keratinocytes. Int. J. Cancer 54 (4), 656–662. 10.1002/ijc.2910540422 7685745

[B43] Szklarczyk D. Gable A. L. Nastou K. C. Lyon D. Kirsch R. Pyysalo S. (2021). The STRING database in 2021: Customizable protein-protein networks, and functional characterization of user-uploaded gene/measurement sets. Nucleic Acids Res. 49 (D1), D605–D612. 10.1093/nar/gkaa1074 33237311 PMC7779004

[B44] Takemoto K. Imai Y. Saito K. Kawasaki T. Carlton P. M. Ishiguro K. I. (2020). Sycp2 is essential for synaptonemal complex assembly, early meiotic recombination and homologous pairing in zebrafish spermatocytes. PLoS Genet. 16 (2), e1008640. 10.1371/journal.pgen.1008640 32092049 PMC7062287

[B45] Tripathi N. Keshari S. Shahi P. Maurya P. Bhattacharjee A. Gupta K. (2020). Human papillomavirus elevated genetic biomarker signature by statistical algorithm. J. Cell. Physiol. 235 (12), 9922–9932. 10.1002/jcp.29807 32537823

[B46] Uhlen M. Fagerberg L. Hallstrom B. M. Lindskog C. Oksvold P. Mardinoglu A. (2015). Proteomics. Tissue-based map of the human proteome. Science 347 (6220), 1260419. 10.1126/science.1260419 25613900

[B47] Winkel K. Alsheimer M. Ollinger R. Benavente R. (2009). Protein SYCP2 provides a link between transverse filaments and lateral elements of mammalian synaptonemal complexes. Chromosoma 118 (2), 259–267. 10.1007/s00412-008-0194-0 19034475

[B48] Wu C. Tuo Y. (2019). SYCP2 expression is a novel prognostic biomarker in luminal A/B breast cancer. Future Oncol. 15 (8), 817–826. 10.2217/fon-2018-0821 30511892

[B49] Yamaguchi K. Mishima K. Ohmura H. Hanamura F. Ito M. Nakano M. (2018). Activation of central/effector memory T cells and T-helper 1 polarization in malignant melanoma patients treated with anti-programmed death-1 antibody. Cancer Sci. 109 (10), 3032–3042. 10.1111/cas.13758 30066977 PMC6172076

[B50] Yang F. De La Fuente R. Leu N. A. Baumann C. McLaughlin K. J. Wang P. J. (2006). Mouse SYCP2 is required for synaptonemal complex assembly and chromosomal synapsis during male meiosis. J. Cell Biol. 173 (4), 497–507. 10.1083/jcb.200603063 16717126 PMC2063860

[B51] Yang F. Gell K. van der Heijden G. W. Eckardt S. Leu N. A. Page D. C. (2008). Meiotic failure in male mice lacking an X-linked factor. Genes Dev. 22 (5), 682–691. 10.1101/gad.1613608 18316482 PMC2259036

[B52] Yang Y. Zheng H. Zhan Y. Fan S. (2019). An emerging tumor invasion mechanism about the collective cell migration. Am. J. Transl. Res. 11 (9), 5301–5312. 31632511 PMC6789225

[B53] Yoshihara K. Shahmoradgoli M. Martinez E. Vegesna R. Kim H. Torres-Garcia W. (2013). Inferring tumour purity and stromal and immune cell admixture from expression data. Nat. Commun. 4, 2612. 10.1038/ncomms3612 24113773 PMC3826632

[B54] Yu G. Li F. Qin Y. Bo X. Wu Y. Wang S. (2010). GOSemSim: an R package for measuring semantic similarity among GO terms and gene products. Bioinformatics 26 (7), 976–978. 10.1093/bioinformatics/btq064 20179076

[B55] Yu G. Wang L. G. Han Y. He Q. Y. (2012). clusterProfiler: an R package for comparing biological themes among gene clusters. OMICS 16 (5), 284–287. 10.1089/omi.2011.0118 22455463 PMC3339379

[B56] Zhou Y. Tian Q. Gao H. Zhu L. Zhang Y. Zhang C. (2022). Immunity and extracellular matrix characteristics of breast cancer subtypes based on identification by T helper cells profiling. Front. Immunol. 13, 859581. 10.3389/fimmu.2022.859581 35795662 PMC9251002

