# Supplementary material for: Increased expression of SYCP2 predicts poor prognosis in patients suffering from breast carcinoma
Source: Front Genet. 2022 Sep 7;13:922401. doi: 10.3389/fgene.2022.922401 (PMC9491682; doi:10.3389/fgene.2022.922401)

Characteristics	N	HR (95% CI)	P value
T stage			
T1	277	1.62 (0.81−3.25)	0.175
T2	629	1.42 (0.91−2.22)	0.124
T3	139	2.70 (1.15−6.33)	0.023
T4	35	0.77 (0.25−2.37)	0.653
N stage			
N0	514	2.42 (1.30−4.49)	0.005
N1	358	1.41 (0.85−2.35)	0.181
N2	116	1.21 (0.49−2.97)	0.681
N3	76	0.56 (0.19−1.69)	0.305
M stage			
M0	902	1.46 (1.02−2.10)	0.038
M1	21	1.68 (0.55−5.19)	0.366
Histological type			
Infiltrating Ductal Carcinoma	772	1.50 (1.02−2.20)	0.04
Infiltrating Lobular Carcinoma	205	1.45 (0.63−3.33)	0.378

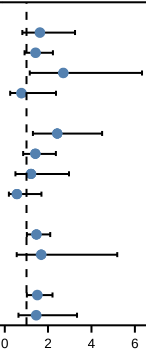

Supplement: Supplementary file 2 [file DataSheet11.zip › Sup-S6-Figure 9+table 5/9H μú«μ₧ùσ¢╛.pdf]
