# Supplementary material for: Increased expression of SYCP2 predicts poor prognosis in patients suffering from breast carcinoma
Source: Front Genet. 2022 Sep 7;13:922401. doi: 10.3389/fgene.2022.922401 (PMC9491682; doi:10.3389/fgene.2022.922401)
Supplement: Supplementary file 2 [file DataSheet11.zip › Sup-S6-Figure 9+table 5/9I Nomogramσ¢╛.pdf]

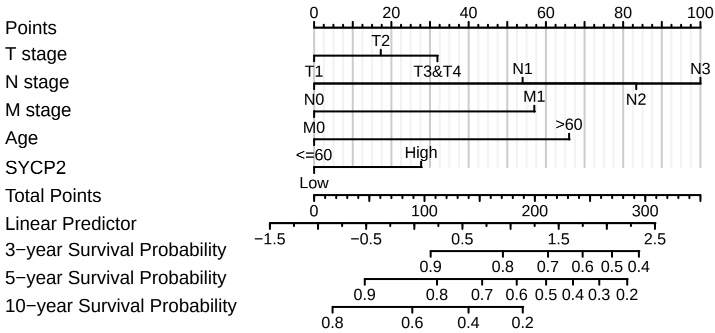
Points
T stage
N stage
M stage
Age
SYCP2
Total Points
Linear Predictor
3−year Survival Probability
5−year Survival Probability
10−year Survival Probability
0 20 40 60 80 100
T2
T1
T3&T4
N1
N3
N0
N2
M1
M0
>60
<=60
High
Low
0 100 200 300
−1.5 −0.5 0.5 1.5 2.5
0.9 0.8 0.7 0.6 0.5 0.4
0.9 0.8 0.7 0.6 0.5 0.4 0.3 0.2
0.8 0.6 0.4 0.2
