# Supplementary material for: Increased expression of SYCP2 predicts poor prognosis in patients suffering from breast carcinoma
Source: Front Genet. 2022 Sep 7;13:922401. doi: 10.3389/fgene.2022.922401 (PMC9491682; doi:10.3389/fgene.2022.922401)
Supplement: Supplementary file 2 [file DataSheet11.zip › Sup-S6-Figure 9+table 5/σìò-σñÜσ¢áτ┤áCoxσ¢₧σ╜Æ.docx]

| Characteristics | Total(N) | Univariate analysis | |  | Multivariate analysis | |
| --- | --- | --- | --- | --- | --- | --- |
|  |  | Hazard ratio (95% CI) | P value |  | Hazard ratio (95% CI) | P value |
| T stage | 1079 |  |  |  |  |  |
| T1 | 276 | Reference |  |  |  |  |
| T2 | 629 | 1.332 (0.887-1.999) | 0.166 |  | 1.117 (0.452-2.758) | 0.810 |
| T3&T4 | 174 | 1.953 (1.221-3.123) | **0.005** |  | 2.590 (0.852-7.875) | 0.093 |
| N stage | 1063 |  |  |  |  |  |
| N0 | 514 | Reference |  |  |  |  |
| N1 | 357 | 1.956 (1.329-2.879) | **<0.001** |  | 1.448 (0.675-3.108) | 0.342 |
| N2 | 116 | 2.519 (1.482-4.281) | **<0.001** |  | 1.278 (0.355-4.609) | 0.707 |
| N3 | 76 | 4.188 (2.316-7.574) | **<0.001** |  | 2.960 (0.818-10.711) | 0.098 |
| M stage | 922 |  |  |  |  |  |
| M0 | 902 | Reference |  |  |  |  |
| M1 | 20 | 4.254 (2.468-7.334) | **<0.001** |  | 3.211 (0.509-20.234) | 0.214 |
| Pathologic stage | 1059 |  |  |  |  |  |
| Stage I | 180 | Reference |  |  |  |  |
| Stage II | 619 | 1.697 (0.985-2.922) | 0.057 |  | 0.811 (0.244-2.695) | 0.732 |
| Stage III | 242 | 2.962 (1.664-5.273) | **<0.001** |  | 1.486 (0.251-8.795) | 0.662 |
| Stage IV | 18 | 11.607 (5.569-24.190) | **<0.001** |  |  |  |
| Race | 993 |  |  |  |  |  |
| Asian | 60 | Reference |  |  |  |  |
| Black or African American | 180 | 1.525 (0.463-5.024) | 0.488 |  |  |  |
| White | 753 | 1.325 (0.420-4.186) | 0.631 |  |  |  |
| Histological type | 977 |  |  |  |  |  |
| Infiltrating Ductal Carcinoma | 772 | Reference |  |  |  |  |
| Infiltrating Lobular Carcinoma | 205 | 0.827 (0.526-1.299) | 0.410 |  |  |  |
| PR status | 1029 |  |  |  |  |  |
| Negative | 342 | Reference |  |  |  |  |
| Positive | 687 | 0.732 (0.523-1.024) | 0.068 |  | 0.931 (0.425-2.039) | 0.858 |
| ER status | 1032 |  |  |  |  |  |
| Negative | 240 | Reference |  |  |  |  |
| Positive | 792 | 0.712 (0.495-1.023) | 0.066 |  | 0.444 (0.194-1.013) | 0.054 |
| HER2 status | 715 |  |  |  |  |  |
| Negative | 558 | Reference |  |  |  |  |
| Positive | 157 | 1.593 (0.973-2.609) | 0.064 |  | 1.028 (0.576-1.834) | 0.926 |
| Age | 1082 |  |  |  |  |  |
| <=60 | 601 | Reference |  |  |  |  |
| >60 | 481 | 2.020 (1.465-2.784) | **<0.001** |  | 3.142 (1.885-5.238) | **<0.001** |
| SYCP2 | 1082 |  |  |  |  |  |
| Low | 540 | Reference |  |  |  |  |
| High | 542 | 1.594 (1.152-2.204) | **0.005** |  | 1.653 (1.002-2.725) | **0.049** |
