# Supplementary material for: Increased expression of SYCP2 predicts poor prognosis in patients suffering from breast carcinoma
Source: Front Genet. 2022 Sep 7;13:922401. doi: 10.3389/fgene.2022.922401 (PMC9491682; doi:10.3389/fgene.2022.922401)
Supplement: Supplementary file 2 [file DataSheet11.zip › Sup-S6-Figure 9+table 5/σƒ║τ║┐Φ╡äμûÖΦí¿.docx]

| Characteristic | levels | Low expression of SYCP2 | High expression of SYCP2 | p |
| --- | --- | --- | --- | --- |
| n |  | 541 | 542 |  |
| T stage, n (%) | T1 | 131 (12.1%) | 146 (13.5%) | 0.509 |
|  | T2 | 317 (29.4%) | 312 (28.9%) |  |
|  | T3 | 71 (6.6%) | 68 (6.3%) |  |
|  | T4 | 21 (1.9%) | 14 (1.3%) |  |
| N stage, n (%) | N0 | 257 (24.2%) | 257 (24.2%) | 0.256 |
|  | N1 | 186 (17.5%) | 172 (16.2%) |  |
|  | N2 | 60 (5.6%) | 56 (5.3%) |  |
|  | N3 | 30 (2.8%) | 46 (4.3%) |  |
| M stage, n (%) | M0 | 448 (48.6%) | 454 (49.2%) | 0.806 |
|  | M1 | 11 (1.2%) | 9 (1%) |  |
| Pathologic stage, n (%) | Stage I | 85 (8%) | 96 (9.1%) | 0.570 |
|  | Stage II | 320 (30.2%) | 299 (28.2%) |  |
|  | Stage III | 116 (10.9%) | 126 (11.9%) |  |
|  | Stage IV | 10 (0.9%) | 8 (0.8%) |  |
| Age, n (%) | <=60 | 325 (30%) | 276 (25.5%) | 0.003 |
|  | >60 | 216 (19.9%) | 266 (24.6%) |  |
| Histological type, n (%) | Infiltrating Ductal Carcinoma | 394 (40.3%) | 378 (38.7%) | 0.009 |
|  | Infiltrating Lobular Carcinoma | 83 (8.5%) | 122 (12.5%) |  |
| PR status, n (%) | Negative | 189 (18.3%) | 153 (14.8%) | 0.006 |
|  | Indeterminate | 4 (0.4%) | 0 (0%) |  |
|  | Positive | 328 (31.7%) | 360 (34.8%) |  |
| ER status, n (%) | Negative | 146 (14.1%) | 94 (9.1%) | < 0.001 |
|  | Indeterminate | 1 (0.1%) | 1 (0.1%) |  |
|  | Positive | 374 (36.1%) | 419 (40.5%) |  |
| HER2 status, n (%) | Negative | 283 (38.9%) | 275 (37.8%) | 0.802 |
|  | Indeterminate | 5 (0.7%) | 7 (1%) |  |
|  | Positive | 81 (11.1%) | 76 (10.5%) |  |
| Age, meidan (IQR) |  | 56 (47, 66) | 60 (50, 68) | < 0.001 |
