# Supplementary figures and images for: Increased expression of SYCP2 predicts poor prognosis in patients suffering from breast carcinoma
Source: Front Genet. 2022 Sep 7;13:922401. doi: 10.3389/fgene.2022.922401 (PMC9491682; doi:10.3389/fgene.2022.922401)

The expression of SYCP2  
 $\text{Log}_2(\text{FPKM}+1)$

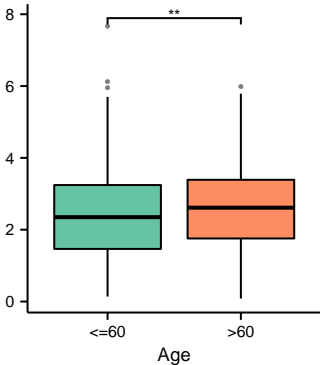

Supplement: Supplementary file 2 [file DataSheet11.zip › Sup-S6-Figure 9+table 5/9A σ╣┤Θ╛ä.pdf]

The expression of SYCP2  
 $\text{Log}_2(\text{FPKM}+1)$

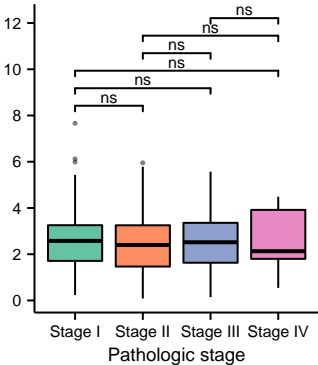

Supplement: Supplementary file 2 [file DataSheet11.zip › Sup-S6-Figure 9+table 5/9B σêåμ£ƒ.pdf]

The expression of SYCP2  
 $\text{Log}_2(\text{FPKM}+1)$

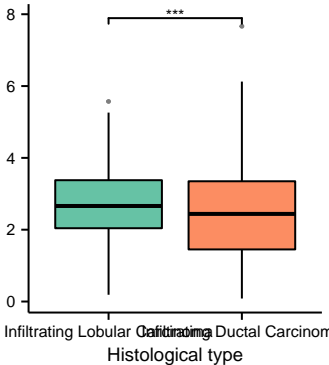

Supplement: Supplementary file 2 [file DataSheet11.zip › Sup-S6-Figure 9+table 5/9C σêåσ₧ï.pdf]

The expression of SYCP2  
 $\text{Log}_2(\text{FPKM}+1)$

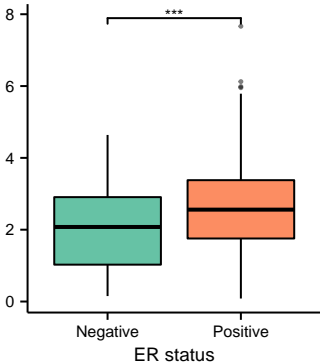

Supplement: Supplementary file 2 [file DataSheet11.zip › Sup-S6-Figure 9+table 5/9D ER.pdf]

The expression of SYCP2  
 $\text{Log}_2(\text{FPKM}+1)$

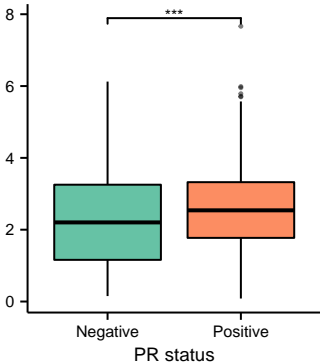

Supplement: Supplementary file 2 [file DataSheet11.zip › Sup-S6-Figure 9+table 5/9E PR.pdf]

The expression of SYCP2  
 $\text{Log}_2(\text{FPKM}+1)$

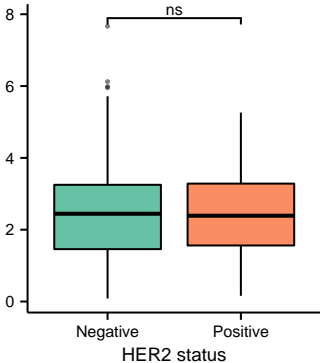

Supplement: Supplementary file 2 [file DataSheet11.zip › Sup-S6-Figure 9+table 5/9F HER2.pdf]

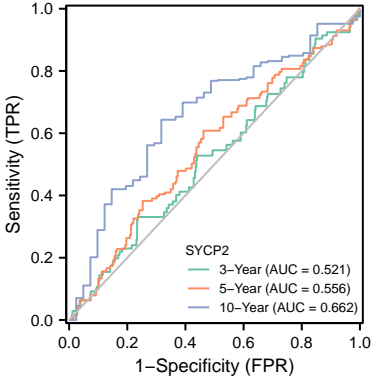

Supplement: Supplementary file 2 [file DataSheet11.zip › Sup-S6-Figure 9+table 5/9G μù╢Θù┤Σ╛¥Φ╡ûROC.pdf]

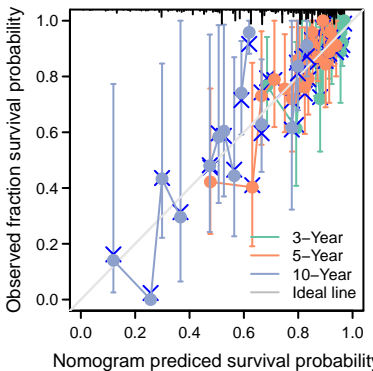

Supplement: Supplementary file 2 [file DataSheet11.zip › Sup-S6-Figure 9+table 5/9J Calibrationσ¢╛.pdf]

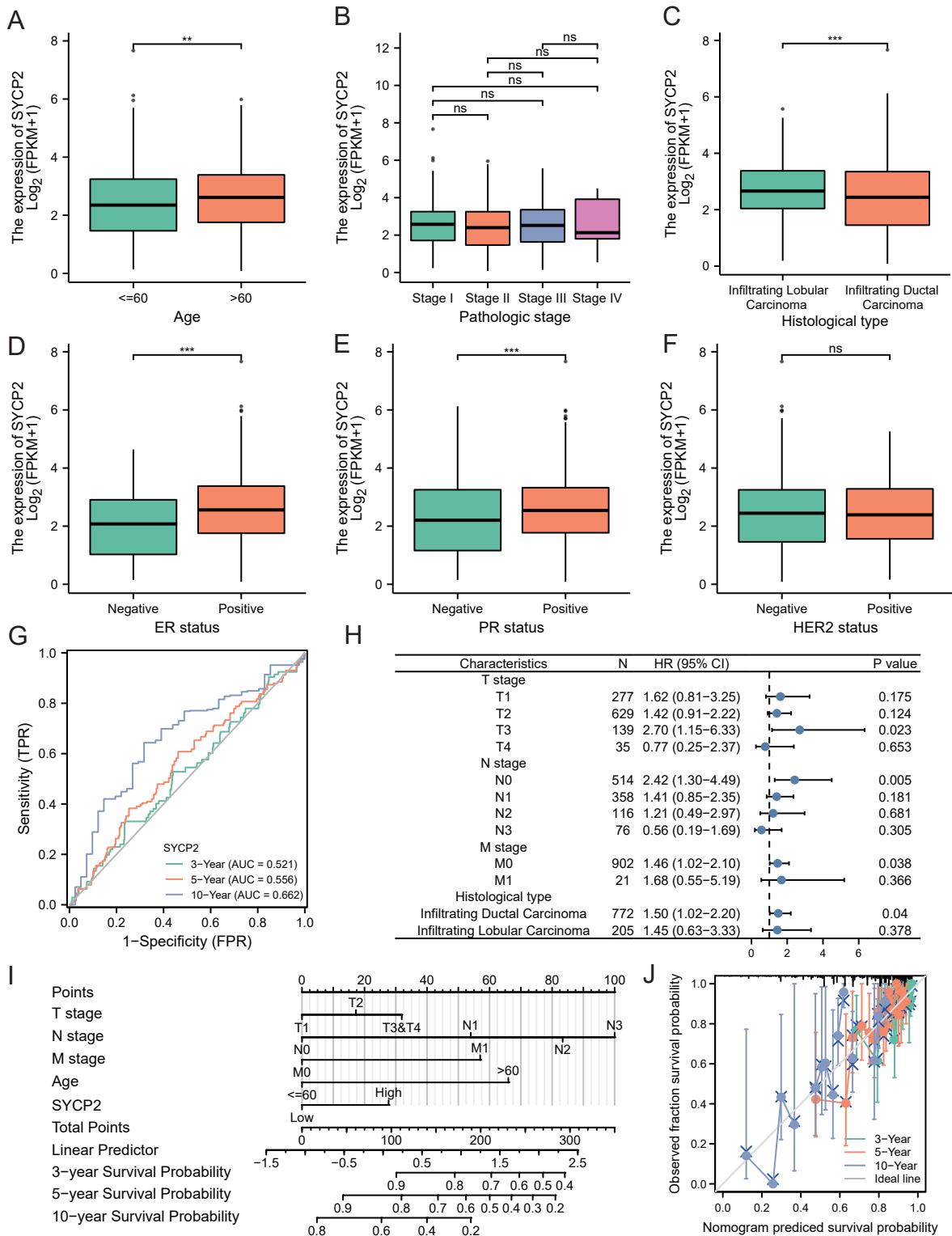

Supplement: Supplementary file 2 [file DataSheet11.zip › Sup-S6-Figure 9+table 5/Figure 9.pdf]
